# A Contingency Framework for the Performance Consequences of Team Boundary Management: A Meta-Analysis of 30 Years of Research

**DOI:** 10.1177/01492063231206107

**Published:** 2023-11-19

**Authors:** Ulrich Leicht-Deobald, Julia Backmann, Thomas A. de Vries, Matthias Weiss, Sebastian Hohmann, Frank Walter, Gerben S. van der Vegt, Martin Hoegl

**Affiliations:** Trinity College Dublin and University of St.Gallen; 9185 University of Münster; University of Groningen; Zeppelin University Friedrichshafen; Apleona Group; Justus-Liebig-University Giessen; University of Groningen; Ludwig-Maximilians-Universität München

**Keywords:** team boundary management, team boundary spanning, team boundary work, team performance, team effectiveness, meta-analysis

## Abstract

Research suggests that teams can greatly enhance their performance through boundary management, which comprises activities that establish, maintain, and regulate linkages with the surrounding environment. However, such performance gains do not materialize equally in all instances, and some teams struggle to benefit from boundary management. Integrating insights from social network and team-level resource allocation theories, we develop a contingency framework that considers the internal organization of a team's boundary management (i.e., the carrier, target, and type of such activities) as a key moderating factor that accounts for the varying effects. To test this framework, we use a meta-analytic approach that synthesizes >30 years of empirical research (i.e., 85 primary studies covering 10,848 teams). Our results show a positive main effect of team boundary management on team performance. Crucially, these performance benefits are more pronounced when the target of boundary management is extraorganizational rather than inside the home organization and when the type of boundary management activities is boundary spanning (e.g., coordination, representation, or information search) rather than boundary strengthening (e.g., buffering, guarding, or sentry activities). Moreover, boundary management is more effective when executed by formal team leaders rather than team members, and our results tentatively suggest that this may reflect differences in effectiveness between leaders and members in boundary strengthening, rather than boundary spanning. Overall, our findings advance theory on team boundary management by clarifying previously ambiguous findings and illustrating how teams can design their boundary management activities to be most effective.

Work teams do not operate in a vacuum. Rather, they must carefully and continuously manage crucial interactions with their surrounding environment (Ancona & Caldwell, 1992a; Marrone, Tesluk, & Carson, 2007). Such *boundary management* comprises a team's activities that establish, maintain, and regulate connections with external parties inside and outside its home organization (Joshi, Pandey, & Han, 2009; Marrone, 2010). These activities may provide teams with vital resources, such as support, knowledge, and information (Marrone, 2010), and enable them to coordinate their efforts with relevant constituents to exploit synergies and avoid conflicts (Davison, Hollenbeck, Barnes, Sleesman, & Ilgen, 2012; de Vries, Hollenbeck, Davison, Walter, & van der Vegt, 2016). Moreover, active boundary management may enable a team to prevent resource losses and keep team members focused on internal tasks (Faraj & Yan, 2009). Generally, therefore, scholars have concluded that a team's boundary management activities should enhance its performance (Bresman, 2010; Haas, 2010; Hoegl, Weinkauf, & Gemuenden, 2004).^1^

Nevertheless, studies have found marked differences in the extent to which teams benefit from boundary management (Choi, 2002; Marrone, 2010). Although these activities yield substantial advantages for some teams (e.g., Cummings, 2004; de Vries, Walter, van der Vegt, & Essens, 2014), others struggle to fully realize these benefits (e.g., Gladstein, 1984; Katz, 1982; Sherman & Keller, 2010). In the latter case, teams may invest considerable effort in establishing and regulating external connections, but they may fail to obtain adequate resources in return. For these teams, boundary management primarily represents a costly, labor-intensive activity that does not substantively improve relevant outcomes (Faraj & Yan, 2009; Gibson & Dibble, 2013; Marrone et al., 2007; Sleep, Bharadwaj, & Lam, 2015). Hence, although boundary management has the potential to promote team performance, the extant literature suggests that these benefits do not materialize equally in all instances.

Within this body of research, previous studies have examined vastly different ways in which a team can organize its boundary management activities. These differences lie in the individuals or groups responsible for a team's boundary management, the individuals or groups targeted through such efforts, and the actual activities performed to implement a team's boundary management (for an overview, see Dey & Ganesh, 2017; Marrone, 2010). Scholars have argued that this variability may account for the diversity and apparent inconsistency of findings (Choi, 2002; Marrone, 2010), although there is considerable debate about the relative advantages and disadvantages associated with different approaches to boundary management (Luciano, DeChurch, & Mathieu, 2018).

In sum, the extensive empirical research on team boundary management has produced diverse and often inconsistent findings, and it has examined substantially different approaches to boundary management. As such, it is difficult to integrate the extant literature into a clear-cut theoretical understanding of how teams effectively organize their boundary management activities (Marrone, 2010). This study uses meta-analytic procedures to address this issue, enabling us to draw from >30 years of empirical research and thus to incorporate a larger sample of teams with more diverse approaches to boundary management than would be possible in a primary study (cf. Gonzalez-Mulé & Aguinis, 2017). Our goal is to examine the diverse approaches to boundary management employed as key contingency factors for the effectiveness of a team's boundary management and, in so doing, to offer a theoretically and empirically sound explanation for the varied performance outcomes observed in prior research.

To provide a systematic understanding of the performance consequences associated with different boundary management approaches, our study integrates insights from social network theory (Park, Grosser, Roebuck, & Mathieu, 2020) and team-based applications of resource allocation theory (Barnes, Hollenbeck, Wagner, DeRue, & Nahrgang, 2008; Porter, Webb, & Gogus, 2010; Porter, Gogus, & Yu, 2011). First, scholars have emphasized that a team's boundary management essentially reflects its efforts to design and organize network connections with external parties (Joshi et al., 2009; Marrone, 2010). Hence, social network concepts are useful for methodically describing a team's diverse boundary management approaches and theoretically deriving our variable selection. Building on prior research (Park et al., 2020), we believe that three network aspects are particularly relevant in this regard: (a) the carrier of a team's boundary management activities, (b) the target of those activities, and (c) the type of boundary management activities.

Second, as noted previously, scholars have argued that teams engage in boundary management to obtain and/or protect critical resources (Ancona & Caldwell, 1992a; Gibson & Dibble, 2013; Marrone, 2010). Hence, we utilize insights from team-level resource allocation theory to describe the potential effectiveness of different boundary management approaches. Depending on a team's particular carrier, target, and type of boundary management, we hold that such activities differ in the extent to which they entail the potential for resource gains and require resource investments (cf. Barnes et al., 2008; Porter et al., 2010). Hence, our conceptual model casts these network characteristics as key contingency factors for the success or failure of a team's boundary management, as illustrated in Figure 1.

**Figure 1:**
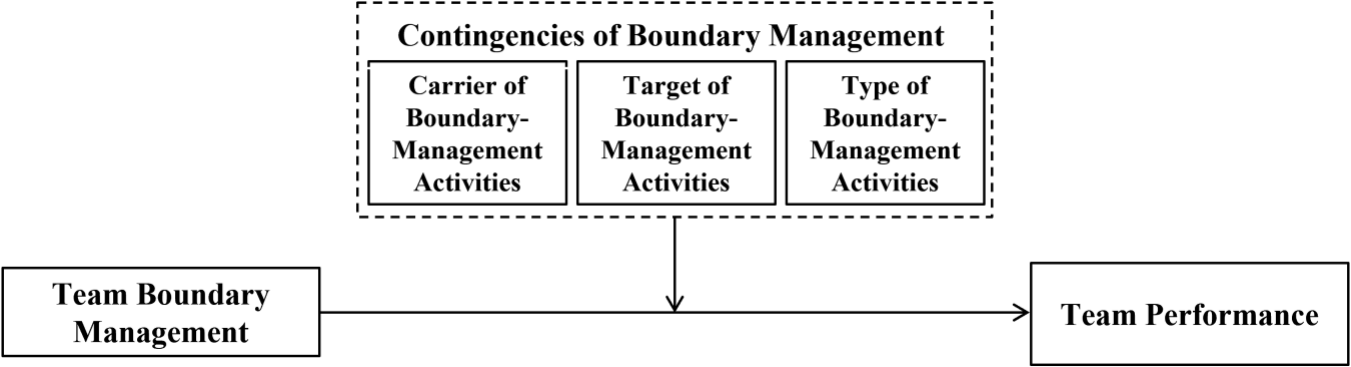
A Contingency Framework of the Team Boundary Management–Performance Link

Our meta-analysis examines this model using a sample of 10,848 teams from 85 primary studies. In so doing, we make important contributions to theory advancement in the team boundary management literature. Beyond offering a systematic, quantitative summary of this mature and complex field of inquiry, this investigation provides new knowledge on *when* a team's boundary management is likely to be effective. As such, it enables a better, theoretically integrated understanding of the diverse findings from prior research on the link between boundary management and team performance. By directly addressing Marrone's (2010: 931) call for studies that explore “at a finer-grained level how teams can most effectively carry out critical boundary spanning processes,” our examination thus offers novel insights into the successful design of team boundary management. Relatedly, we contribute to team-based resource allocation theory by extending its logic to examine how external activities may affect team performance. Past studies using this theoretical approach have primarily examined how team activities draw from internal resources without considering the possibility that such activities may enable teams to access additional, external resources. We expand this perspective to conceptualize boundary management as a critical activity that may strengthen a team's resource base and thus improve its performance outcomes, and we aim to illustrate new, previously unidentified boundary conditions for the effectiveness of this activity type.

## Theory and Hypotheses Development

### Team Boundary Management and Team Performance: State of the Research

Team boundary management encompasses all of a team's activities aimed at establishing, nurturing, and/or regulating linkages with its external environment and managing resource exchanges with external constituents inside or outside its home organization (Ancona, 1990; Ancona & Caldwell, 1992a; Faraj & Yan, 2009). These activities include coordinating joint task accomplishment, lobbying for external resources, soliciting outside information and/or support, and protecting the team from undue interference (Ancona, 1990; Ancona & Caldwell, 1992a).

Traditionally, scholars have argued that such boundary management yields pronounced team performance benefits (Dey & Ganesh, 2017; Marrone, 2010). This argument is based on the notion that the superior coordination and additional resources obtained through such activities improve the efficiency and effectiveness of a team's work processes as well as its task accomplishment (Marrone, 2010). Numerous empirical studies have reported positive linkages between a team's boundary management and various indicators of team performance, including outcome quality (e.g., Keller, 2001; Menon, Jaworski, & Kohli, 1997; Wu, Huang, Jiang, Klein, & Liu, 2020), innovativeness and creativity (e.g., Akgün, Dayan, & Di Benedetto, 2008; Chang & Cho, 2008; Edmondson, 2003), and efficiency (e.g., Denison, Hart, & Kahn, 1996; Keller, 2001; Scott, 1997). In contrast, other researchers have emphasized that boundary management yields tangible problems and costs that may attenuate or even reverse its performance advantages (Choi, 2002). A core insight underlying this perspective is that boundary management is effortful and time-consuming, which may distract the team from internal activities and goals (Carbonell & Rodríguez Escudero, 2019; Faraj & Yan, 2009; Gibson & Dibble, 2013; Sleep et al., 2015; Tsai, 2002). Accordingly, a number of empirical studies have revealed nonsignificant (e.g., Haas, 2006; Lewis, Welsh, Dehler, & Green, 2002; Sawyer, Guinan, & Cooprider, 2010) or even negative (e.g., Atuahene-Gima, 2003; Hansen, 1999; Katz, 1982; Kianto, 2011) relationships between boundary management and team performance.

Given these inconsistent main effects, it is not surprising that theorists have highlighted the potential relevance of moderating variables for the team boundary management–performance relationship (Choi, 2002). Notably, however, a seminal literature review on boundary management has identified a dearth of research on moderating influences (Marrone, 2010), and few empirical studies have examined such interactive relations since the publication of that review (Dey & Ganesh, 2017).^2^ Hence, although the link between team boundary management and performance is likely to hinge on relevant contingency factors, empirical work on this issue is still in its infancy, which has prompted repeated calls for additional research in this regard (Dey & Ganesh, 2017; Marrone, 2010). The present study uses meta-analytic procedures to address those calls.

### Diverse Approaches to Boundary Management: A Social Network Perspective

Our choice to use a meta-analytic research design is based on the observation that the inconsistent findings of extant studies and the high number of such studies offer a unique opportunity to address scholarly calls for the examination of moderated relationships (e.g., Faraj & Yan, 2009; Gibson & Dibble, 2013). The boundary management approaches covered in this stream of literature may constitute important yet unexamined contingency factors for boundary management's performance consequences (Choi, 2002), and meta-analytic methods are well suited for uncovering such contingencies in an existing body of research (Aguinis, Beaty, Boik, & Pierce, 2005; Gonzalez-Mulé & Aguinis, 2017).

A meta-analytic approach should start with a theory-driven description of relevant team boundary management approaches to enable the systematic identification of potential moderators. As noted earlier, social network theory is useful in this regard because, at its core, boundary management focuses on creating and regulating a team's network connections with the external environment (Joshi et al., 2009; Marrone, 2010). Hence, we believe that key constructs from social network theory can be fruitfully applied to the description of a team's distinct boundary management approaches. In particular, team-based applications of social network theory suggest that three elements are crucial for understanding the way that a team organizes its internal and external network connections (Humphrey & Aime, 2014; Park et al., 2020):

*Ego:* the individuals or groups within the team implementing relevant network interactions

*Alters:* the individuals or groups outside the team toward whom such interactions are directed

*Ties:* the interactive ego-alter connections themselves (Park et al., 2020).

This tripartite classification is directly applicable to prior research on team boundary management. For example, with regard to ego attributes, studies have examined different *carriers* of a team's activities. Some studies have described boundary management as the exclusive responsibility of formal leaders as representatives of their teams (e.g., Davison et al., 2012; Richter, West, Van Dick, & Dawson, 2006; Tröster, Mehra, & van Knippenberg, 2014). Others have framed boundary management as a responsibility shared by team members regardless of their hierarchical positions (e.g., de Vries, van der Vegt, Bunderson, Walter, & Essens, 2022; de Vries et al., 2014; Marrone et al., 2007). With respect to alter attributes, research has examined different *targets* of a team's boundary management efforts. Some studies have focused on boundary management between constituents in the same organization (e.g., Ancona & Caldwell, 1992a; Sherman & Keller, 2010), while others have adopted a broader, extraorganizational perspective (e.g., Gopal & Gosain, 2010; Mishra & Shah, 2009). Finally, with regard to tie attributes, prior research has examined a variety of boundary management *types* (i.e., specific activities or strategies, such as coordination, representation, or buffering). Broadly, these types can be categorized as activities aimed at either connecting the team more closely with external parties (i.e., boundary spanning; de Vries et al., 2014; Haas, 2010; Lewis et al., 2002; Mishra & Shah, 2009) or minimizing external influences and distractions (i.e., boundary strengthening; Ancona & Caldwell, 1988; Faraj & Yan, 2009).

On this basis, our conceptual model distinguishes among the carrier (i.e., formal leaders vs. team members), the target (i.e., within or outside a team's home organization), and the type (i.e., boundary strengthening vs. boundary spanning) of a team's boundary management as key moderators of the performance consequences of such activities (Figure 1). In the following, we explore the roles of these contingency factors and develop hypotheses by integrating resource allocation theory.

### A Resource Allocation Framework of Team Boundary Management's Effectiveness

#### Theoretical background

Although a social network perspective is useful for systematically describing different approaches to team boundary management, it does not allow for specific predictions regarding how these approaches influence associated performance effects. As noted earlier, prior research has suggested that boundary management may affect team performance by shaping the resources available for task accomplishment. Specifically, boundary management may facilitate effective and efficient task execution by providing teams with external support, expertise, and information, but it may also be a labor-intensive activity that prevents team members from devoting their time and attention to internal tasks.^3^ Hence, we extend our previous considerations with insights from team-based applications of resource allocation theory (Barnes et al., 2008; Porter et al., 2010) to consider how different boundary management approaches may affect the time and attentional resources that teams have available for task accomplishment, thereby influencing team performance.

Organizational researchers have widely applied resource allocation theory (e.g., Bergeron, 2007; Smillie, Yeo, Furnham, & Jackson, 2006), and they have investigated how teams’ dedication of time and attention to various activities influences their performance (e.g., Nielsen, Bachrach, Sundstrom, & Halfhill, 2012; Porter et al., 2010, 2011). A core premise of this theoretical perspective, as applied to organizational work teams, is that members’ time and attention are finite resources (Nielsen et al., 2012; Porter et al., 2011); hence, investments in a specific activity come at the expense of other actions (Barnes et al., 2008). Moreover, the ultimate performance consequences of any team activity hinge on the degree to which the resources gained from that activity compensate for the invested resources (Bamberger & Levi, 2009). As noted earlier, team-level resource allocation theory has traditionally focused on intrateam processes (e.g., members’ backing up behavior, feedback, or internal coordination; Bamberger & Levi, 2009; Barnes et al., 2008; Porter et al., 2010, 2011) and conceptualized teams as closed systems without access to outside resources (e.g., Barnes et al., 2008; Mitchell, Boyle, Nicholas, Maitland, & Zhao, 2016; Porter et al., 2010). We extend this theory toward external boundary management interactions and their associated resource implications.

Specifically, we assert that a team's boundary management entails the potential for resource gains and resource losses. On one hand, such activities may enable teams to protect their finite internal resources from external demands and/or supplement those resources with external inputs (e.g., by securing outside assistance or new information; Quick & Feldman, 2014). On the other, boundary management requires the investment of finite resources, such as members’ effort and attention (Ancona & Caldwell, 1988; Marrone et al., 2007). Team-level resource allocation theory suggests that the balance between these resource gains and resource investments is critical for determining the performance consequences of a team's boundary management efforts. Moreover, we propose that this balance critically hinges on the approach that a team uses to manage its boundaries—that is, on the carrier, target, and type of a team's boundary management. As we outline here, each of these approaches entails distinct potential for resource gains and requires specific resource investments, which uniquely shape boundary management's overall performance implications.

#### Baseline hypothesis: The main effect of team boundary management on team performance

Drawing from our resource allocation argumentation, we first introduce our baseline hypothesis, which predicts a positive net effect of team boundary management on team performance. Specifically, we generally expect a positive balance between resource investments in and resource gains from team boundary management (Dey & Ganesh, 2017; Marrone, 2010). Through boundary management, a team can acquire critical resources, such as information from extraorganizational experts (Gopal & Gosain, 2010); supplier, customer, and client feedback (Maruping, 2006; Mishra & Shah, 2009); top management support (Ancona & Caldwell, 1992a); and technical help from other teams in the home organization (Hoegl et al., 2004). Furthermore, boundary management can help protect important team resources by, for example, safeguarding vital information (Guinan, Cooprider, & Faraj, 1998) and preserving members’ time, energy, and attention (Sawyer et al., 2010; Somech & Khalaili, 2014). This general argumentation is supported by a range of literature that has found a positive relationship between team boundary management and team performance (Dey & Ganesh, 2017; Joshi et al., 2009; Marrone, 2010), even though such benefits may be dampened or strengthened depending on how boundary management is executed (Choi, 2002).

*Hypothesis 1:* Team boundary management is positively associated with team performance.

#### Carrier of team boundary management

With regard to the carrier of a team's boundary management, we expect that boundary management activities will yield superior performance benefits when jointly executed by a team's members as compared with a formal team leader's efforts. A key reason for this is that members’ boundary management has the potential to trigger greater resource gains. Team members’ boundary management is likely to create a relatively large number of ties between the focal team's members and the team's external environment (Oh, Chung, & Labianca, 2004; Park et al., 2020). This may enable the team to access a large amount of diverse resources through numerous communication and coordination channels, thus enriching the team's resource base in a rather comprehensive manner (Bouty, 2000; Reagans, Zuckerman, & McEvily, 2004). Moreover, the resources gained through members’ boundary management likely target the team's specific operational needs and thus should be highly useful for the team's goal attainment, given that the members executing such activities should be intimately familiar with key operational and technical requirements of the team's tasks (Keller, 2001; Sawyer et al., 2010).

In terms of required resource investments, team members’ and a team leader's boundary management may both yield distinct advantages and disadvantages. For instance, if boundary management activities are shared among team members, each individual can limit his or her involvement and thus may avoid excessive external distractions (Marrone et al., 2007). In addition, team members can fill in for one another, flexibly distributing boundary management responsibilities to match the team's current workflows and minimize the burden on any one individual (DeChurch & Marks, 2006). At the same time, engaging in boundary management can distract team members from their primary tasks, with potential efficiency costs for daily task accomplishment (Cummings, 2004; Guinan et al., 1998). The latter issue is less problematic for a formal team leader's boundary management, as such activities are unlikely to substantively disrupt the other team members’ task focus and efficiency (Davison et al., 2012). At the same time, a team leader may become overburdened by his or her external boundary management duties (Marrone et al., 2007), potentially aggravating the accomplishment of internal leadership tasks that are crucial to the team's functioning (e.g., managing the team's internal processes; Marks, DeChurch, Mathieu, Panzer, & Alonso, 2005; Richter et al., 2006).

In sum, this reasoning suggests that the boundary management activities performed by team members may yield greater resource gains than those performed by a formal leader. At the same time, both these approaches require specific resource investments that will likely incur comparable costs for the team. When considered collectively, the net effects for team performance are expected to be more positive when team members, rather than a team leader, handle boundary management.

*Hypothesis 2:* The positive relationship between team boundary management and team performance is stronger for boundary management activities executed by team members than for those executed by the formal team leader.

#### Target of team boundary management

Although all boundary management activities are, by definition, directed toward entities outside a focal team, scholars have drawn a sharp distinction between boundary management focused on targets inside or outside a team's home organization (e.g., Poole & Contractor, 2012; Zaccaro, Dubrow, Torres, & Campbell, 2020). Drawing from our resource allocation framework, we expect the boundary management cost-benefit ratio to be more advantageous and thus yield stronger performance benefits when such activities are directed toward extraorganizational rather than intraorganizational targets.

This argumentation builds on the notion that managing boundaries with parties beyond the home organization may permit a team to gather vital information about broad environmental trends that might otherwise be missed or obtained much later (Geletkanycz & Hambrick, 1997). More generally, extraorganizational boundary management can provide a team with novel and diverse information, perspectives, and resources that are not readily available within the home organization, thereby considerably extending the team's resource pool (Cross & Cummings, 2004). In contrast, the knowledge and information acquired through intraorganizational boundary management are more likely to resemble the resources already available to the team because the targets share similar goals, perspectives, and working methods (Teigland & Wasko, 2003). Hence, although intraorganizational boundary management may yield resource benefits (e.g., enable better coordination between organizational teams; Hoegl et al., 2004), we expect those benefits to be qualitatively different and, in many cases, less pronounced than the benefits gained through extraorganizational efforts. Along these lines, Menon and Pfeffer (2003) have argued that organizational members attach more value to knowledge from extraorganizational sources because such knowledge is more likely to be new and unique and can therefore decisively improve the team's performance potential.

With respect to resource investments, boundary management directed at targets outside, rather than inside, a team's home organization can be more costly and effortful due to, for example, the lower accessibility and likelihood of responsiveness from external targets (Albers, Wohlgezogen, & Zajac, 2016; Johnson & Johnston, 2004). Moreover, extraorganizational interactions may suffer from incompatible identities, conflicting cultures, or differing worldviews (Carlile, 2004; Hultink, Talke, Griffin, & Veldhuizen, 2011) and from the need to prevent undue resource leakages (de Vries et al., 2022; Ryu, McCann, & Reuer, 2018). These problems are less pronounced for intraorganizational boundary management, as actors and targets are more likely to share compatible goals, assumptions, and approaches to work (Hansen, 2002; Lewis et al., 2002). Nevertheless, easier access to intraorganizational boundary management partners may also carry unique costs. Research on collaborative overload suggests that a team's internal boundary management efforts often lead to numerous requests for information and support from other teams, which require substantial investments of time and energy from the focal team (Cross, Rebele, & Grant, 2016; Cross & Thomas, 2009). Hence, the net resource investments required for intra- and extraorganizational boundary management may be comparable.

In sum, this reasoning suggests that extraorganizational boundary management holds greater potential for resource gains than intraorganizational boundary management, while both approaches require comparable resource investments. Consistent with prior research (e.g., Rosenkopf & Nerkar, 2001; Sanders, 2007), we expect both approaches to boundary management to eventually trigger performance benefits. Overall, we expect the cost-benefit ratio to be more favorable for extraorganizational than intraorganizational boundary management. As compared with the resources obtained through intraorganizational boundary management, the unique resources obtained through extraorganizational boundary management should be more valuable to the team and thus more easily outweigh the investments required for their acquisition.

*Hypothesis 3*: The positive relationship between team boundary management and team performance is stronger for boundary management activities that are directed toward extraorganizational rather than intraorganizational targets.

#### Type of team boundary management

Finally, we expect boundary management to be more beneficial when it aims at spanning rather than strengthening team boundaries. Boundary-spanning efforts connect a team to external constituents with the explicit aim of obtaining additional resources (de Vries et al., 2014; Haas, 2010; Mishra & Shah, 2009). Research has shown that such activities offer the potential for pronounced resource gains, as they can enable a team to garner external inputs and extend its resource pool (Faraj & Yan, 2009; Gibson & Dibble, 2013; Haas, 2006). This type of boundary management may, for instance, offer an effective means of acquiring expert knowledge, information, and tangible support from other individuals or teams (Ancona & Caldwell, 1988, 1992a). In contrast, boundary strengthening aims to minimize external influences and avoid associated distractions by, for example, restricting outside parties’ access to the team or preventing members from reaching out to external constituents (Ancona & Caldwell, 1988; Davison et al., 2012; de Vries et al., 2014). Such activities can protect the team's internal resources, thereby enabling members to focus their time and attention on core tasks and limiting undesirable information leakages (Dey & Ganesh 2017; Gibson & Dibble, 2013). However, as a team cannot gain access to new resources through boundary strengthening, this type of boundary management does not offer the potential for substantial resource gains.

At the same time, the resource investments associated with boundary spanning and boundary strengthening are likely comparable. Boundary spanning requires actors to invest in meeting with external constituents (Ancona, 1990), coordinating activities (de Vries et al., 2014), accommodating external parties’ perspectives and needs (Faraj & Yan, 2009), and nurturing important relationships (Ancona, 1990; Carmeli & Azeroual, 2009). Likewise, strengthening a team's boundaries requires the investment of resources to buffer the team against information requests (Faraj & Yan, 2009), monitor external relationships (Ancona & Caldwell, 1992b), and ensure that the team does not unwittingly release sensitive information (Guinan et al., 1998). Hence, both types of boundary management require the time, attention, and effort of the actors within a team, and we do not see a clear basis to assert that one type will incur greater or fewer costs in this regard.

In sum, while boundary spanning and boundary strengthening require comparable resource investments, only boundary spanning entails the potential for additional, substantial resource gains. Therefore, the net utility should be more pronounced for boundary spanning than for boundary strengthening.

*Hypothesis 4:* The positive relationship between team boundary management and team performance is stronger for boundary-spanning activities than for boundary-strengthening activities.

## Methods

### Literature Search

We used meta-analytic methods to test the hypotheses. To identify relevant primary studies, we first searched scholarly databases for studies on team boundary management (i.e., ABI/Inform, Business Source Premier, ProQuest Dissertations, PsycINFO, ISI Web of Science, and Scopus). We scanned titles, keywords, and abstracts for the search terms *team* or *group* in conjunction with keywords such as *boundary management*, *boundary spanning*, *boundary work*, *ambassador*, *coordination*, *guard*, *scout*, *sentry, external communication*, *information gathering*, *bridging relationships*, *cross-boundary relationships*, and *intergroup ties*. The cutoff date for inclusion in the meta-analysis was December 31, 2021, while the beginning of our sampling period was 1981—the point at which the first empirical work on team boundary management activities was published (Katz & Tushman, 1981).

We then manually searched the titles and abstracts of all articles published in major outlets for team and group research since 1981 to identify any articles on team boundary management that we might have missed in our keyword search. These outlets included the *Journal of Management*, the *Journal of Applied Psychology*, the *Academy of Management Journal*, *Small Group Research*, *Organization Science*, *Organizational Behavior and Human Decision Processes*, and the *Journal of Organizational Behavior*. We also performed backward and forward searches on conceptual reviews of the literature on team boundary management (Dey & Ganesh, 2017; Marrone, 2010) and the articles identified in the first two steps to ensure that we did not overlook any relevant studies. We posted requests for unpublished papers and data on team boundary management on relevant academic listservs, including those associated with the Academy of Management's divisions of organizational behavior, human resources, strategic management, and technology and innovation management, as well as the American Marketing Association's ELMAR Listserv. Finally, to locate other unpublished studies, we perused the programs of pertinent conferences held from 2014 to 2020, including the Annual Meeting of the Academy of Management, the Interdisciplinary Network for Group Research Conference, and the Conference of the Society for Industrial and Organizational Psychology.

Thereafter, we examined all the identified sources (1,299 publications, manuscripts, and data sets) to exclude those unsuitable for our meta-analysis because they did not (a) examine team boundary management with some aspect of team performance, (b) contain quantitative findings (e.g., theoretical or qualitative studies), or (c) report correlation coefficients or provide sufficient information to calculate such coefficients (Lipsey & Wilson, 2001; this also applied to studies exclusively reporting beta coefficients; see Roth, Le, Oh, Van Iddekinge, & Bobko, 2018). Moreover, given our theoretical interest in the team-level performance implications of boundary management, we excluded studies that exclusively focused on the organizational or individual levels of analysis. Furthermore, we did not include studies on new venture teams because their structure and governance differ substantively from common organizational teams (e.g., venture teams represent the entire organization and their members are typically founders with equity interests; Kamm, Shuman, Seeger, & Nurick, 1990; Sirén, He, Wesemann, Jonassen, Grichnik, & von Krogh, 2020). Finally, we did not include alliance teams (e.g., Drach-Zahavy, 2011; Pao, Wu, & Lee, 2020) because members of such teams originate from different organizations; thus, the distinction between internal team activities and boundary management is blurred.

**Figure 2:**
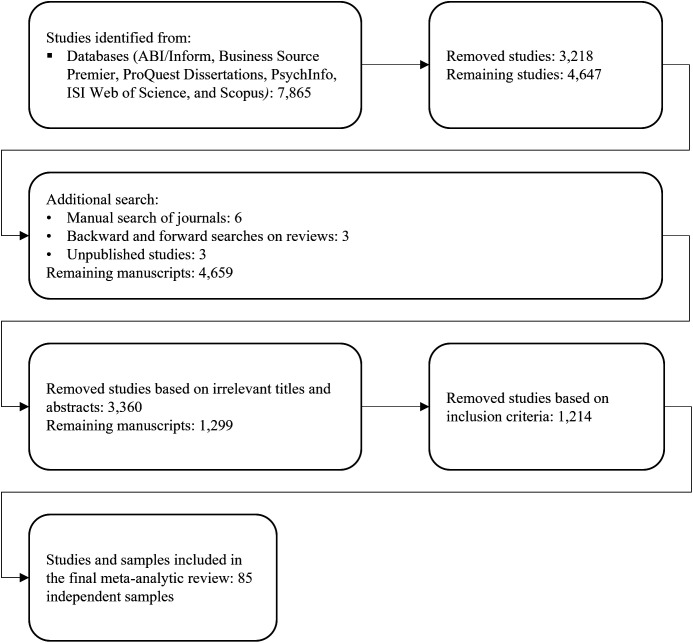
PRISMA Flowchart

We reviewed all remaining studies for overlapping samples. If the same data appeared in more than one study, we retained only one of the respective correlations. Some studies split samples or examined two or more samples. If there was no data overlap, we treated these samples as independent. Based on these decision criteria, our final data set contained 85 independent samples covering 10,848 teams. Figure 2 summarizes the literature search and process in a PRISMA flow diagram (Benjamin, Kepes, & Bushman, 2018).

### Coding and Variables

We developed a detailed manual to establish relevant rules and decision norms for coding the primary studies. The first five authors coded the studies. To enhance consistency, the coders met multiple times to discuss this process and its outcomes. In an initial round, each individual independently coded the same five studies. Subsequently, the coders jointly discussed their results to calibrate their coding procedures and refine the manual. All further studies were coded independently by two individuals (Hunter & Schmidt, 2004) with satisfactory interrater agreement (83%). A key reason for disagreements was that the sample and measure descriptions in the primary studies varied widely and were often rather vague, making it difficult to establish clear-cut coding rules that covered all eventualities. The interrater agreement was calculated by the preconsensus discussion. However, the research team discussed any discrepancies until 100% agreement was reached.

First, we coded all bivariate correlations between any boundary management and team performance variables reported in the sample studies as a basis for assessing Hypothesis 1. Second, we coded the theoretical moderators (i.e., carrier, target, and type of boundary management) to determine whether the overall main effect of boundary management was altered depending on how such activities were organized (Hypotheses 2-4). Finally, we coded several methodological moderators and covariates to partial out the effects of these variables and thus enable more valid hypotheses tests. In the following, we describe the exact procedures for coding the moderators and covariates.

#### Theoretical moderators

We coded the *carrier* of a team's boundary management based on whether these activities were limited to the team's formal leader (i.e., team managers or supervisors; coded 0; Richter et al., 2006; Sleep et al., 2015) or involved the team's members (coded 1; e.g., Kim, Min, & Cha, 1999; Marrone et al., 2007). Furthermore, we coded whether the *target* of a team's boundary management was located within (coded 0) or outside (coded 1) a focal team's home organization. Examples of activities aimed at intraorganizational targets include cross-functional coordination with other teams at the same hierarchical level as well as attempts to influence top management (e.g., Denison et al., 1996; Sleep et al., 2015). Examples of extraorganizational boundary management include gathering advice from outside experts or obtaining feedback from customers (e.g., Mishra & Shah, 2009). While a finer-grained coding for the target of boundary management would have allowed us to measure the exact distance between a focal team and the target, the majority of the sample studies did not provide sufficient information.

Finally, with regard to the *type* of boundary management, we coded the activities as boundary strengthening (coded 0) when they aimed to shield a team from external disturbances (i.e., sentry or boundary-buffering activities) or avoid the leakage of internal information (i.e., guard activities; Ancona & Caldwell, 1988). We coded activities as boundary spanning (coded 1) when they aimed to coordinate with external entities, search for information, exchange knowledge/resources with outside parties, or represent the team in interactions with stakeholders (Kratzer, Leenders, & Van Engelen, 2008).

A short illustration may help demonstrate our moderator coding. For example, Edmondson (1999) used a boundary management measure labeled “team leader external relationships.” This measure used the stem “The team leader . . .” and continued with sample items such as “takes initiatives to help us get the support and cooperation we need from other parts of the company” and “helps us get the resources from other parts of the organization that our team needs to do its work.” We coded this measure to represent leaders’ (i.e., carrier) intraorganizational (i.e., target) boundary spanning (i.e., type). As another example, Rojas et al. (2021) used a measure labeled “external communication” that was completed by team members and aggregated to the team level, with sample items including “I share information with members from other teams within the organization” and “I am in touch with people of the organization external to my team.” We coded this measure to represent members’ (i.e., carrier) intraorganizational (i.e., target) boundary spanning (i.e., type). If we could not unequivocally assign a boundary management activity to a specific carrier, target, or type, we coded the information as missing. Appendix 1 provides a more detailed overview of the relevant coding criteria, with further sample studies.

#### Methodological moderators and control variables

We considered several methodological moderators that might influence the relationship between boundary management and team performance. First, the level of *specificity* in the measurement of boundary management activities varies among studies. While some studies broadly assess boundary management as work-related interactions with external parties without explaining the specific character of this interaction (e.g., frequency of external communication), many use a more detailed approach that includes the purpose or content of the activities (e.g., coordination, general information search, or sentry activities). To capture these differences, we included a dummy variable that indicated whether studies measured general (0) or specific (1) boundary management activities.

Second, the performance consequences of boundary management may differ across *performance criteria*. Prior team-level meta-analyses broadly distinguished two important performance dimensions: team efficiency and team effectiveness (Bell, 2007; Dey & Ganesh, 2017; Greer, de Jong, Schouten, & Dannals, 2018). Team efficiency, which refers to the process of task-related team functioning (Beal, Cohen, Burke, & McLendon, 2003), comprises indicators such as a team's adherence to budgets and schedules as well as its overall efficiency (e.g., Ancona & Caldwell, 1992a). Team effectiveness refers to a team's output, task accomplishment, and goal attainment (Beal et al., 2003), including such indicators as product quality, innovation and creativity, stakeholder satisfaction, and market success (e.g., Leenders & Voermans, 2007; Somech & Khalaili, 2014). Meta-analytic reviews of team research often distinguish such aspects to accommodate the richness of different performance criteria (e.g., de Jong, Dirks, & Gillespie, 2016: Sivasubramaniam, Liebowitz, & Lackman, 2012). On this basis, we included a dummy code to indicate whether a performance variable was related to efficiency (0) or effectiveness (1).^4^

Third, we included a dummy variable to indicate whether studies used the *same measurement source* to assess the independent and dependent variables (coded as 1; e.g., subjective member ratings of boundary management and team performance; Hirst & Mann, 2004) or independent or objective data sources for these variables (coded as 0; e.g., Hansen, 2002). We incorporated this covariate to account for potential common-source or method biases in the observed relationships (Crampton & Wagner, 1994; Podsakoff, MacKenzie, & Podsakoff, 2012).

Fourth, the sample studies featured two approaches to operationalizing boundary management. Some studies used network-based measures. These studies typically asked respondents to identify the external partners with whom they engaged in boundary management and then calculated a team's overall boundary management on this basis (e.g., de Vries et al., 2014; Tsai, 2002). Other studies used Likert-type questionnaire items that asked respondents to indicate how frequently they engaged in boundary management in general with larger groups of partners (i.e., how often respondents interacted with other teams without asking them to identify specific teams or members), and they used these data to derive team-level boundary management scores (e.g., Ancona & Caldwell, 1992a; Faraj & Yan, 2009). Despite these differences, studies using either approach generally employed similar items and thus captured largely equivalent constructs. For example, using a network measure, Hansen et al. (2005: 782) employed the item “Looking back over the last year, are there any persons in your subsidiary from whom you regularly sought information and advice to help your project work?” Similarly, using a Likert-type measure, Ancona and Caldwell (1992a: 641; 1992b) asked respondents to rate the extent to which they “collect technical information/ideas from individuals outside of the team.” Nevertheless, to account for possible differences arising from the two approaches, we included a dummy variable that reflected whether a study used a *network-based* (coded 1) or *Likert* (coded 0) measure for boundary management. Notably, when all studies that employed network-based measures of boundary management were omitted, the results from this reduced sample (269 effect sizes from 72 studies) were consistent with our full sample findings, as reported in the following section. These supplementary findings are depicted in Appendix 6.^5^

Fifth, some studies asked the team leaders to rate the boundary management items (e.g., Hansen, 2002; Mishra & Shah, 2009), while others asked the team members to do so (e.g., Lewis et al., 2002; Wong, 2004). Hence, we included a *respondents* dummy, which indicated whether team leaders (0) or members (coded 1) rated the boundary management items.

Finally, like many team-level constructs, boundary management can be measured through the aggregation of the individual members’ activities or through team-referent (i.e., referent-shift) scale items (Chan, 1998). Meta-analytic research has shown that the effects of a team-level construct may differ depending on its aggregation model (Wallace, Edwards, Paul, Burke, Christian, & Eissa, 2016). Hence, we included an *aggregation models* dummy for primary studies using team member ratings of boundary management, which indicated whether these studies used referent-shift items (coded 1; e.g., Bresman, 2005) or not (coded 0; e.g., Marrone et al., 2007).

In addition to these boundary management–specific moderators, we included six study-level controls. We controlled for the *industry* in which a focal team was active, which distinguished manufacturing-based (coded 0) and service-based (coded 1) industries. Boundary management may be particularly essential in service-based industries because customer contact is vital in this context (Knight, Menges, & Bruch, 2018). Furthermore, we controlled for the type of organization to which the focal teams belonged by differentiating large *multinational companies* (coded 1) and other organizations (coded 0). Intraorganizational targets of boundary management might be less accessible and more distant yet potentially offer more diverse insights in multinational companies than in smaller organizations (Klueter & Monteiro, 2017). Moreover, we controlled for *R&D teams* (coded 1) and non-R&D teams (coded 0) because R&D teams may be particular dependent on boundary management activities (Elkins & Keller, 2003).

We also included a control variable to capture the *type of team tasks*. In line with prior team meta-analyses (De Dreu & Weingart, 2003; de Wit, Greer, & Jehn, 2012), we built on McGrath's (1984) group task circumplex model and identified project tasks, decision-making tasks, and production-planning tasks as proxies for the extent of task complexity, uncertainty, and routineness, respectively. Project tasks reflect creative and innovative tasks; as such, they are the most uncertain, most complex, and least routinized. In contrast, production-planning tasks are focused on task execution according to predefined performance standards. They therefore represent the most certain, least complex, and most routine task type. Decision-making tasks fall in the middle of this continuum—they represent tasks that require team members to reach agreement about a certain strategic direction or solution. In addition, we created dummy variables for the three task categories. Moreover, we added a mixed category for studies reporting that teams worked on a variety of tasks (coded 0). Furthermore, we controlled for *study setting* (i.e., field study [0] vs. laboratory study [1]), as induced boundary management in a laboratory context may differ from naturally occurring boundary management activities (Maloney, Bresman, Zellmer-Bruhn, & Beaver, 2016). Moreover, to control for different reporting habits in research outlets, we included a variable reflecting *publication status*, which indicated whether a study was published in a peer-reviewed journal (1) or not (0). Finally, we included the *year of publication*, as the role of team boundary management may have changed over time (Dey & Ganesh, 2017).

### Meta-Analytic Procedures

#### Bivariate analysis

To test Hypothesis 1, we examined the overall relationship between boundary management and team performance irrespective of the carrier, target, or type of boundary management activities. We followed Hunter and Schmidt's (2004) meta-analysis approach to obtain an estimate of the relevant population correlation based on the raw correlations from the primary studies. In so doing, we used random-effects meta-analysis because it allows for the possibility that parameters vary across studies and for the estimation of variability among studies (Schmidt, 2015). As is common practice, we used observed Pearson correlation coefficients as our bivariate effect size metric, and we corrected for sampling errors by weighting sample sizes when calculating mean correlations.

Furthermore, we calculated the effect size after correcting for measurement error in the boundary management and team performance measures by dividing the individual effect sizes by the product of the square root of the reliability estimates of the correlated variables (Hunter & Schmidt, 2004). Following previous team-level meta-analyses (e.g., De Jong et al., 2016; Greer et al., 2018), we used interrater reliability (i.e., ICC_2_) to correct correlations for measurement error. ICC_2_ captures the reliability of team-level mean scores (Bliese, 2000) and accounts for inconsistency across raters as a key source of unreliability (Courtright, Thurgood, Stewart, & Pierotti, 2015). When boundary management or performance was measured objectively, we assumed perfect reliability and imputed a reliability of 1; when no ICC_2_ value was reported, we imputed the average reliability estimate for those variables (Hunter & Schmidt, 2004).^6^ Importantly, when a sample reported multiple operationally distinct variables that were categorized as a similar boundary management construct or team performance variable and these correlations showed similar categorizations in terms of the control variables, we combined them into a single correlation coefficient using linear composites to ensure that the effect sizes were independent (Schmidt, 2015).

In addition to the estimated, corrected true population correlation, we report the 95% confidence interval (95% CI) and the 80% credibility interval to indicate effect size variability. The confidence interval indicates the possible amount of sampling error in the *point estimate* of a population correlation, while the credibility interval indicates the *range* of a population correlation across studies after correcting for sampling error (Gonzalez-Mulé & Aguinis, 2017). Furthermore, to assess data heterogeneity, we report the *Q* statistic (i.e., the chi-square test of homogeneity) and *I*^2^ (i.e., the percentage of variance due to artifacts). A significant *Q* statistic or an *I*^2^ <75% suggests the potential presence of moderators in a meta-analytic correlation (Gonzalez-Mulé & Aguinis, 2017).

#### Meta-regression

We employed multilevel meta-analytic regression analyses to test Hypotheses 2 to 4. Specifically, we applied a variance-known multilevel regression analysis using full maximum likelihood estimation with robust standard errors and effect sizes corrected for measurement error to explain the heterogeneity of correlations between team boundary management and team performance (Van den Noortgate, López-López, Marín-Martínez, & Sánchez-Meca, 2014). In other words, we analyzed how the proposed moderators affected the relationship between boundary management and team performance. Therefore, the dependent variable in our regression models is represented by the correlation between boundary management and team performance. We chose a multilevel approach, which is particularly suitable for accounting for the nested nature of our data (Hox, 2010). In line with Van den Noortgate and colleagues' (2014) recommendations, we applied a three-level regression model using Mplus 8.4 (Muthén & Muthén, 1998–2017) to account for the fact that multiple effect sizes originated from the same studies and thus to avoid inflated type I error rates (see also Holmes, Jiang, Avery, McKay, Oh, & Tillman, 2021). In this regard, Level 1 represents the variance within the effect sizes (modeled as variance known); Level 2, the level of the effect sizes; and Level 3, the study level. We calculated the variance estimate in the variance-known models by applying the large-sample approximation suggested by Borenstein (2009).^7^

## Results

### Boundary Management and Team Performance: Overall Relationship

Hypothesis 1 predicts a positive association between boundary management and team performance. As depicted in Table 1, the bivariate meta-analytic correlation coefficient is positive and its 95% CI does not include zero (ρ* *= 0.39 [0.33, 0.45]). This supports Hypothesis 1. Furthermore, the *Q* statistic is significant (*Q *= 785.95, *p *< .001), and the percentage of between-study variance attributable to artifacts is substantially <75% (*I*^2 ^= 11%). This indicates substantial heterogeneity among individual studies, which warrants a moderator analysis.

**Table 1:** Results for the Main Effect of Team Boundary Management on Team Performance (Test of Hypothesis 1) and Subgroup Analysis of Theoretical Moderators

Variable	*k*	*n*	*r*		ρ					Q***	*I*^2^, %
			Mean	*SD*	Mean	*SD*	95% CI	80% CV	*SE*		
H1^a^	85	10,848	0.26	0.18	0.39	0.27	.33, .45	.05, .73	0.03	785.95	11
Carrier of boundary management											
Leader	13	1,312	0.25	0.14	0.39	0.21	.26, .52	.12, .66	0.06	59.28	22
Members	36	4,459	0.25	0.20	0.37	0.30	.26, .48	−.01, .75	0.06	398.24	9
Target of boundary management											
Intraorganizational	49	6,238	0.23	0.16	0.34	0.24	.27, .41	.03, .64	0.04	360.79	13
Extraorganizational	19	2,216	0.26	0.15	0.40	0.23	.29, .51	.11, .69	0.05	115.56	16
Type of boundary management											
Boundary strengthening	9	842	0.17	0.20	0.31	0.35	.06, .55	−.13, .75	0.13	100.22	8
Boundary spanning	57	5,917	0.29	0.19	0.43	0.28	.35, .50	.07, .78	0.04	451.90	12

*Note: k = *number of independent studies; *n *= cumulative sample size; *r = *sample size–weighted mean observed correlation; ρ* *= estimated mean true score correlation; 95% CI = 95% confidence interval; 80% CV = credibility interval; *Q *= Cochran's homogeneity test statistic; *I^2 ^= *scale-free index of heterogeneity.

a Hypothesis 1: Relationship between team boundary management and team performance.

***Each *Q* statistic, *p* < .001 (2-tailed).

### Moderators of the Boundary Management–Team Performance Link

Table 1 illustrates bivariate subgroup analyses for the proposed moderators, and Table 2 shows descriptive statistics and bivariate correlations for all the variables in our subsequent meta-analytic regression models. Of the 295 effect sizes from the primary studies, 129 (44%) include the carrier; 183 (62%), the target; and 217 (74%), the type of boundary management.^8^

**Table 2:** Descriptive Statistics and Correlations for Meta-Regression

Variable	Mean	*SD*	Effect Sizes, *k*		1	2	3	4	5	6	7	8	9	10	11	12	13	14	15	16	17	18	19
1: Relationship between team boundary management and team performance^a^	0.32	0.34	295																				
**Controls: study level**																							
2: Industry ^b^	0.40	0.49	88 (0)	59 (1)	.05																		
3: MNC ^c^	0.18	0.38	242 (0)	53 (1)	−.05	.32***																	
4: R&D team ^d^	0.41	0.49	174 (0)	121 (1)	−.03	−.61***	−.28***																
5: Project team tasks ^e^	0.62	0.49	113 (0)	182 (1)	−.11^†^	−.63***	−.14*	.60***															
6: Decision-making team tasks ^f^	0.19	0.39	239 (0)	56 (1)	.08	.51***	−.09	−.40***	−.61***														
7: Production-planning team tasks ^g^	0.02	0.14	289 (0)	6 (1)	.09	−.08	.00	−.12*	−.18**	−.07													
8: Mixed team tasks ^h^	0.17	0.38	245 (0)	50 (1)	.02	.33***	.28***	−.30***	−.57***	−.22***	−.07												
9: Study setting ^i^	0.05	0.23	279 (0)	16 (1)	−.02	.18*	−.11^†^	−.20***	−.30***	.49***	−.03	−.11^†^											
10: Publication status ^j^	0.86	0.34	40 (0)	255 (1)	−.02	.20*	.16**	−.31***	−.21***	.12*	.06	.13*	.09										
11: Year of publication	2,007.92	8.09	295		.14*	.56***	−.01	−.18**	−.31***	.37***	.00	−.01	.05	−.17**									
**Methodological moderators: effect size level**																							
12: Specificity of boundary management ^k^	0.73	0.45	80 (0)	215 (1)	.04	.34***	−.03	−.10	−.04	.10^†^	−.02	−.03	.11^†^	−.13*	.22***								
13: Type of team performance criterion ^l^	0.16	0.37	247 (0)	48 (1)	−.11^†^	−.17*	.03	.10^†^	.18**	−.19**	−.06	.00	−.11^†^	−.07	−.11^†^	.02							
14: Same measurement source ^m^	0.41	0.49	175 (0)	120 (1)	.26***	.15^†^	−.05	−.02	−.09	.07	−.02	.03	−.20***	.19**	.05	−.02	−.20***						
15: Network measure ^n^	0.06	0.24	269 (0)	17 (1)	−.16**	.23**	.18**	−.06	−.11^†^	.00	−.04	.16**	.04	.10^†^	−.07	−.21***	−.07	−.09					
16: Aggregation model ^o^	0.21	0.41	193 (0)	52 (1)	−.18**	−.25**	.06	.15*	−.04	.09	.17**	−.12^†^	.35***	.23***	−.48***	−.10	−.04	−.22***	.41***				
17: Respondents ^p^	0.79	0.41	42 (0)	157 (1)	−.02	.16	.03	−.14*	.07	−.16*	.07	.04	−.02	−.14^†^	−.24***	.11	.12^†^	−.24***	.01	.09			
**Theoretical moderators: effect size level**																							
18: Carrier of boundary management ^p^	0.78	0.41	28 (0)	101 (1)	−.16^†^	.31^†^	−.18*	−.01	.25**	−.41***	.09	.07	−.06	−.29***	−.21*	.15	.21*	−.08	−.02	−.05	.77***		
19: Target of boundary management ^q^	0.25	0.43	138 (0)	45 (1)	.12^†^	−.33**	−.18*	.40***	.32***	−.13^†^	−.09	−.23**	−.18*	−.35***	.19**	−.16*	.04	.13^†^	−.13^†^	−.29***	−.17^†^	.12	
20: Type of boundary management ^r^	0.88	0.33	27 (0)	190 (1)	.25***	.26**	.13*	−.13^†^	−.27***	.20**	.05	.13^†^	.10	.10	.01	−.04	−.02	.17*	.07	.03	−.12	−.09	−.05

*Note: k = *85 (independent studies). Effect sizes: *k* = number of correlations meta-analyzed (numbers per moderator value are shown in parentheses). MNC = multinational company; R&D = research and development.

a 0 = manufacturing based, 1 = service based.

b 0 = no MNC, 1 = MNC.

c 0 = no R&D team, 1 = R&D team.

d 0 = no project team tasks, 1 = project team tasks.

e 0 = no decision-making team tasks, 1 = decision-making team tasks.

f 0 = no production-planning team tasks, 1 = production-planning team tasks.

g 0 = no mixed team tasks, 1 = mixed team tasks.

h 0 = field study, 1 = laboratory study.

i 0 = unpublished, 1 = published.

j 0 = specific boundary management, 1 = general boundary management.

k 0 = efficiency-related performance criterion, 1 = effectiveness-related performance criterion.

l 0 = independent data sources, 1 = same subjective data source for independent and dependent variables.

m 0 = Likert scale measure, 1 = network measure.

n 0 = referent-shift consensus model, 1 = direct consensus model.

o 0 = formal leader, 1 = team members.

p 0 = intraorganizational, 1 = extraorganizational.

q 0 = boundary strengthening, 1 = boundary spanning.

r Effect sizes are based on corrected effect sizes (ρ).

† *p *< .10.

*p < .05.

**p < .01.

***p < .001 (2-tailed).

As shown, the bivariate results suggest that the type of boundary management is positively related to the boundary management–team performance effect sizes (*r *= .25, *p *< .001) such that the positive link between boundary management and performance is stronger for boundary spanning (ρ* *= 0.43, 95% CI [0.35, 0.50]) than boundary strengthening (ρ* *= 0.31, 95% CI [0.06, 0.55]). Moreover, we found a positive and marginally significant correlation between the target of boundary management and the boundary management–team performance effect sizes (*r *= .12, *p *= .095), with this positive relationship being slightly more pronounced for extraorganizational targets (ρ* *= 0.40, 95% CI [0.29, 0.51]) than intraorganizational targets (ρ* *= 0.34, 95% CI [0.27, 0.41]). Finally, there is a negative and marginally significant correlation between the target of boundary management and the boundary management–team performance effect sizes (*r *= –.16, *p *= .064). Contrary to our expectations, this indicates slightly weaker effect sizes for team members’ boundary management (ρ* *= 0.37, 95% CI [0.26, 0.48]) than for leaders’ boundary management (ρ* *= 0.39, 95% CI [0.26, 0.52]), although the difference is very small in this initial analysis.

Crucially, these bivariate analyses do not consider the simultaneous effects of the theoretical moderators and other control variables, while the multivariate meta-regression analysis described here does. Hence, the bivariate results should be interpreted with caution. In fact, scholars have noted that meta-regression helps disentangle and identify effects that otherwise may go unnoticed in bivariate analyses, especially when studying two or more moderators concurrently (Geyskens et al., 2009; Steel & Kammeyer-Mueller, 2002).

Table 3 presents the results of the meta-regression analyses used to formally test Hypotheses 2 to 4. We first entered the control variables and methodological moderators in Model 1 and then added the theoretical moderators proposed in Hypotheses 2 to 4 in Model 2.

**Table 3:** Meta-Analytic Regression Results: Tests of Hypotheses 2-4

Variable^b^	Boundary Management and Team Performance^a^					
	Model 1: Controls Only		Model 2: Research Model		Model 3: Research Model Including Carrier's Spanning	
	γ	*SE*, *p*	γ	*SE*, *p*	γ	*SE*, *p*
**Level 3: Between studies**						
Controls						
Industry	.06	.09, .513	.07	.09, .480	.06	.09, .540
MNC	−.01	.09, .894	−.04	.08, .596	−.04	.08, .653
R&D team	−.02	.08, .821	−.07	.08, .423	−.07	.08., .420
Project task	.09	.22, .687	.12	.21, .591	.10	.19, .604
Decision-making task	−.10	.10, .335	−.20	.11, .084	−.20	.11, .079
Production task	−.05	.10, .580	−.03	.10, .740	−.05	.10, .637
Study setting	−.07	.13, .586	−.02	.15, .902	−.02	.14, .885
Publication status	.01	.10, .911	−.01	.11, .947	.01	.11, .904
Year of publication	.00	.00, .996	.00	.00, .318	.00	.00, .323
**Level 2: Between effect sizes**						
Methodological moderators						
Boundary management specificity	.06	.05, .172	.06	.05, .226	.07	.04, .118
Team performance criterion	−.04	.05, .364	−.04	.04, .398	−.03	.05, .529
Same measurement source	.10	.05, .028	.11	.04, .009	.10	.04, .017
Network measure	−.28	.17, .108	−.27	.16, .097	−.25	.16, .100
Aggregation model	.06	.15, .697	.10	.13, .439	.10	.12, .421
Respondents	.06	.05, .248	.15	.05, .002	.11	.04, .009
Theoretical moderators						
Carrier: H2			−.26	.11, .016	−.32	.10, .001
Target: H3			.09	.05, .046	.09	.04, .015
Type: H4			.18	.07, .006	.16	.06, .007
Carrier's spanning					.25	.09, .003
**Level 1: Variance within effect sizes**	.06	.02, .001	.04	.01, .001	.03	.01, .013
Overall number of observations						
Independent studies	85		85		85	
Effect sizes	295		295		295	
Intraclass coefficient	.34		.34		.34	
Model deviance	5,202.89		5,668.86		5,720.29	

*Note:* Maximum likelihood estimation with robust *SE* and exact *p* value. Entries corresponding to the moderating variables are estimations of the random effects (γ). MNC = multinational company; R&D = research and development.

a Effect sizes are based on corrected correlations (ρ).

b Coding: industry (0 = manufacturing based, 1 = service based); MNC (0 = no MNC, 1 = MNC); R&D team (0 = no R&D team, 1 = R&D team); project team tasks (0 = no project team tasks, 1 = project team tasks); decision-making team tasks (0 = no decision-making team tasks, 1 = decision-making team tasks); production-planning team tasks (0 = no production-planning team tasks, 1 = production-planning team tasks); study setting (0 = field study, 1 = laboratory study); publication status (0 = unpublished, 1 = published); boundary management specificity (0 = specific boundary management, 1 = general boundary management); team performance criterion (0 = efficiency-related performance criterion, 1 = effectiveness-related performance criterion); same measurement source (0 = independent data sources, 1 = same subjective data source for independent and dependent variables); network measure (0 = Likert scale measure, 1 = network measure); aggregation model (0 = referent-shift consensus model, 1 = direct consensus model); carrier (0 = formal leader, 1 = team members); target (0 = intraorganizational, 1 = extraorganizational); type (0 = boundary strengthening, 1 = boundary spanning).

#### Study-level control variables and methodological moderators

As shown in Table 3 (Model 1), none of the nine study-level control variables significantly influenced the relationship between boundary management and team performance. Similarly, five of the six effect size methodological moderators did not significantly influence the boundary management–team performance link. However, for same-source measurement, the *p* value was below the .05 threshold (γ* *= 0.10, *SE* = .05, *p *= .028), indicating that the positive relationship between boundary management and team performance is stronger when the same source evaluated the independent and dependent variables rather than different data sources. Overall, these findings indicate that the suggested control variables do not have an important influence on the link between boundary management and team performance.

#### Theoretical moderators

Hypothesis 2 predicts that the positive relationship between boundary management and team performance is accentuated when team members (coded 1) perform those activities and attenuated when boundary management is executed by the formal team leader (coded 0). As shown in Table 3 (Model 2), however, the coefficient for the carrier of boundary management is negative and significant (γ* *= –0.26, *SE* = .11, *p *= .016), indicating that leaders’ boundary management is more effective than team members’ efforts. Hence, Hypothesis 2 is not supported. We return to this unexpected finding in the Supplementary Analyses and Discussion sections.

Hypothesis 3, which concerns the target of a team's boundary management, predicts that the positive relationship between boundary management activities and team performance is stronger for extraorganizational boundary management (coded 1) than for intraorganizational boundary management (coded 0). As shown in Table 3 (Model 2), the coefficient is positive and significant (γ* *= 0.09, *SE* = .05, *p *= .046). Thus, Hypothesis 3 is supported.^9^

Hypothesis 4 proposes that the positive relationship between team boundary management and team performance is stronger for boundary-spanning activities (coded 1) than for boundary-strengthening activities (coded 0). As shown in Table 3 (Model 2), the coefficient for the type of boundary management is positive and significant (γ* *= 0.18, *SE* = .07, *p *= .006). Hence, Hypothesis 4 is supported.^10^

#### Supplementary analyses

To better understand the unexpected findings for Hypothesis 2 (i.e., the moderating effect of carrier), we conducted a post hoc analysis that focused on formal leaders’ versus team members’ boundary-spanning activities, rather than overall boundary management. In our primary studies, nine effect sizes measured leaders’ boundary-spanning activities, and 25 measured members’ boundary-spanning activities. As shown in Table 3 (Model 3), we found a significant coefficient for the carrier's boundary spanning (γ* *= 0.25, *SE *= .09, *p *= .003). Based on this regression equation, the predicted effect size was only slightly larger for leaders’ (*ŷ *= 0.39) rather than members’ (*ŷ *= 0.32) boundary-spanning activities (Appendix 2). Hence, we tentatively conclude that the substantively stronger effect size for leaders’ rather than members’ overall boundary management, as revealed in our main analyses (*ŷ* = 0.39 vs. *ŷ* = 0.13), may be driven primarily by the greater effectiveness of leaders’ versus members’ boundary strengthening rather than boundary spanning.^11^

We further conducted a mediation analysis to test the notion that increased resource levels may explain *why* boundary management can enhance team performance (Barnes et al., 2008). Resource allocation theory generally focuses more on attentional or cognitive resources rather than material or financial (Kanfer & Ackerman, 1989). Only six of our sample studies examined this resource type (Cummings, 2004; Gautam, 1997; Leicht-Deobald & Lam, 2016; Nasim & Iqbal, 2019; van Woerkom & Croon, 2009; Wong, 2004), thereby preventing the use of robust statistical methods for mediation testing (e.g., meta-analytic structural equation modeling; Viswesvaran & Ones, 1995). To gain some preliminary insights, we therefore adopted these studies to calculate the mean bivariate relationship for the link between boundary management and team (attentional) resources. Similar to our main analysis, we corrected this estimate for sample size and measurement error (i.e., interrater reliability, namely ICC_2_; Greer et al., 2018). Moreover, we utilized Mesmer-Magnus and DeChurch's (2009) meta-analytic effect size estimate for the link between team (attentional) resources and team performance. As shown in Appendix 3, team boundary management was substantially correlated with team (attentional) resources (ρ* *= 0.45, *SD *= 0.15) and team performance (ρ* *= 0.39, *SD *= 0.27), and such resources were substantially correlated with team performance as well (ρ* *= 0.42, *SD *= 0.22). Multiplying the estimates for the linkages of boundary management–resources and resources-performance (see MacKinnon, 2008) yielded an indirect effect size estimate of *a* × *b* = 0.19. Although certainly not conclusive, these findings offer initial evidence for the role of team (attentional) resources as a potential explanatory mechanism in the association between boundary management and team performance.

To demonstrate that our results were not bound to a specific method of correcting the reported correlation coefficients, we repeated our meta-regression using the raw correlations, uncorrected for measurement error (Appendix 4). The results of this analysis did not suggest any changes in the direction or implications of the hypothesized relationships, indicating that our results were not biased by the specification of the effect sizes in the regression models.

Moreover, we explored the robustness of our findings by checking whether the inclusion or exclusion of control variables changed our results. We calculated a series of models that included only those control variables that were significantly related to one of the theoretical moderators (i.e., the carrier, target, or type of boundary management; see Appendix 5, Models 1a-1c). Furthermore, we calculated models that included only the controls significantly related to team performance (Appendix 5, Model 2), only the six methodological controls (Appendix 5, Model 3), and no controls (Appendix 5, Model 4). Across all these supplementary analyses, the effects of the three theoretical moderators remained significant and in the same direction as in our main analysis (as a sole exception, the coefficient for boundary management target was marginally significant in Appendix 5, Model 1c). In sum, these robustness checks suggest that our findings are unlikely to be biased by the inclusion or exclusion of specific control variables.

**Figure 3:**
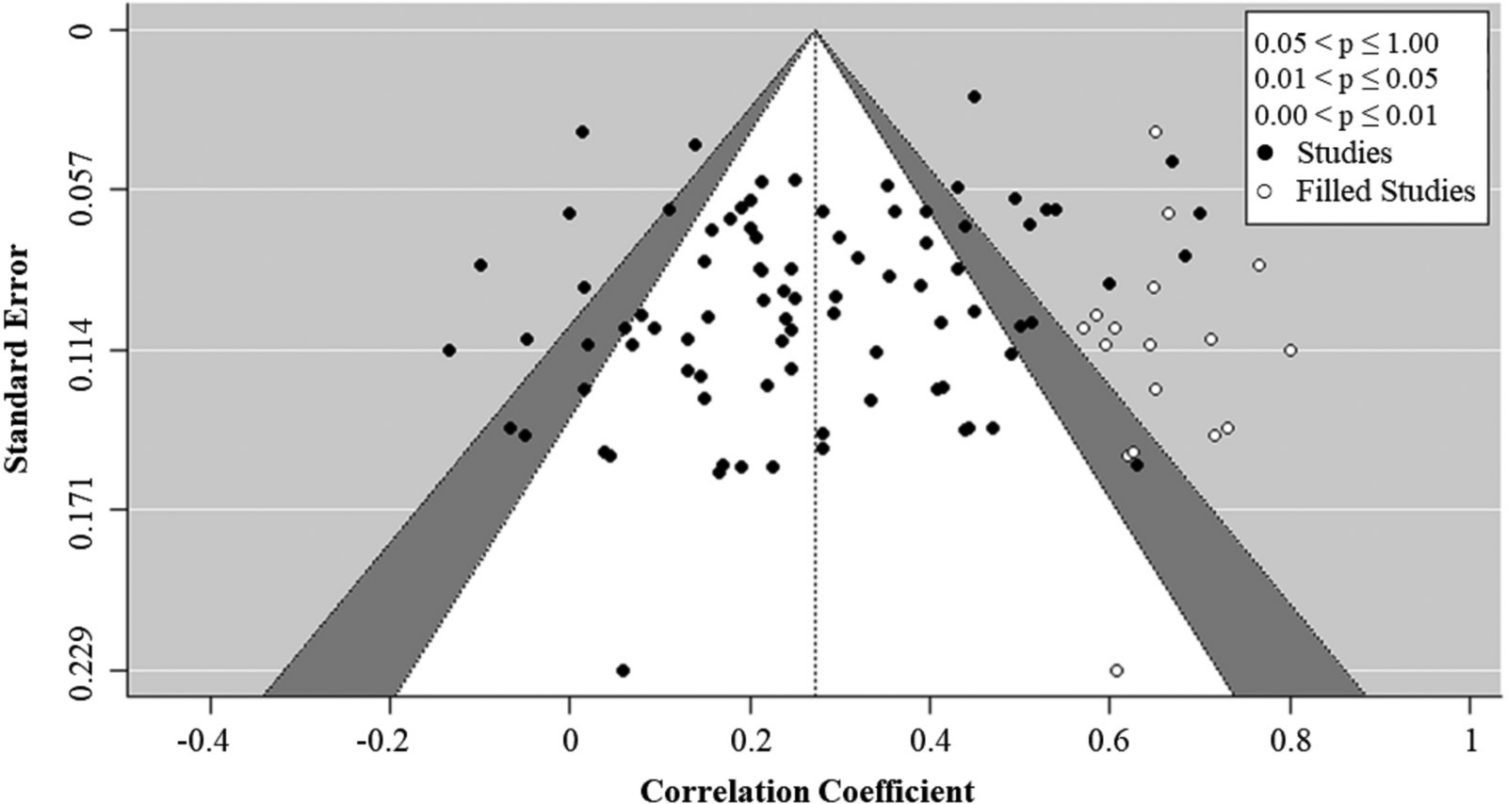
Results of Trim-and-Fill Test

Finally, as guided by the recommendations of Kepes, Banks, McDaniel, and Whetzel (2012), we tested for potential publication biases in the observed overall (study-level) relationships between boundary management and team performance by carrying out a trim-and-fill analysis based on a funnel plot (Figure 3). In this regard, an Egger's test showed significant funnel plot asymmetry (*p *= .035). Moreover, Duval and Tweedie's (2000) trim-and-fill method showed that achieving symmetry required the addition of 17 effect sizes on the right-hand side of the distribution. Hence, these analyses indicate that the true overall relationship between boundary management and team performance may be even more positive than what our meta-analytic point estimate suggests. Notably, this tendency is in the opposite direction of traditional file drawer problems, which suggest that studies with smaller effect sizes are less likely to be published. As Banks, Kepes, and Banks (2012) proposed, a pattern of trim-and-fill results, as observed in our investigation, may result from “true” heterogeneity caused by an uneven distribution of moderating variables across the sample studies. Moreover, Banks et al. (2012) noted that potential publication biases may be negligible when the difference between a meta-analytic mean estimate and a trim-and-fill adjusted estimate is <20%, which is the case in our analysis. As such, we conclude that our meta-analytic findings are unlikely to suffer from file drawer problems or other systematic publication biases.

## Discussion

This research offers new insights into the nature of the relationship between team boundary management and team performance. Drawing from social network and team-level resource allocation theories, our meta-analysis of 85 primary studies reveals that, in general, boundary management enhances a team's performance. Importantly, however, these advantages do not apply equally to all teams. Rather, they crucially depend on how a team organizes its boundary management in terms of the target, type, and carrier of the associated activities.

### Theoretical Implications

Our findings have several relevant implications for the boundary management literature. In particular, they illustrate that the *target* of a team's boundary management plays an essential role in the associated performance consequences. Although previous studies have often focused on teams’ efforts to manage boundaries with relevant parties within the same organization (Ancona & Caldwell, 1992a; Kahn & McDonough III, 1997), some scholars have emphasized the importance of managing a team's relationships with extraorganizational constituents (e.g., Kotha & Srikanth, 2013). Our findings provide support for the latter perspective, indicating that boundary management activities have notable effects when they target actors outside a focal team's home organization. This is not to say that intraorganizational boundary management should be neglected—in fact, our results show that such activities may yield important benefits. Taken together, our findings help to create consensus on the advantages of extraorganizational boundary management, which enables teams to access unique resources and/or avoid undue interference from extraorganizational constituents.

Moreover, our findings clarify the performance consequences associated with different boundary management *types*, a topic of considerable contention in prior research. On one hand, researchers have frequently highlighted the benefits of boundary-spanning efforts aimed at building and maintaining close connections with team-external parties (e.g., Choi, 2002; Joshi et al., 2009; Marrone, 2010). On the other, scholars have not only argued that such boundary spanning can introduce external interference and collaborative overload (Gibson & Dibble, 2013; Luciano, DeChurch, & Mathieu, 2018) but also emphasized the importance of boundary-strengthening activities that shield a team from these issues and safeguard its resources (Wolbers, Boersma, & Groenewegen, 2018; Wu et al., 2020). Our findings illustrate that boundary spanning and boundary strengthening can both enhance a team's performance. Importantly, however, we also show that teams should not expect the same performance advantages from these two types of boundary management. Such benefits are significantly stronger for boundary-spanning activities intended to (a) help coordinate with other entities, (b) represent the team among relevant constituents, and (c) obtain external information than they are for boundary-strengthening activities designed to buffer the team against external interference and protect team resources.

Furthermore, our results shed new light on the *carrier* of a team's boundary management. Contrary to our hypothesis, which was based on relevant theorizing and evidence, our meta-analysis found formal team leaders’ boundary management to be more effective than the boundary management of team members. In both cases, the benefits of such boundary management exceed its costs, with positive consequences for a team's performance. Given the unexpected nature of this finding, we encourage future research that investigates this issue in more detail. Our supplementary analyses indicate that there may be an interaction effect between boundary management carrier and type such that the overall stronger effect of leaders’ (vs. members’) boundary management might be predominantly due to leader-member differences in the role of boundary-strengthening activities rather than boundary-spanning activities. Given the tentative nature of this conclusion, however, additional research is certainly needed to scrutinize its viability.

In addition, our investigation illustrates how integrating a social network perspective with insights from resource allocation theory opens new conceptual perspectives for research on the relationship between boundary management and team performance as well as its boundary conditions. Relatedly, our theoretical framework extends previous team-level applications of resource allocation theory, which have often implicitly assumed that teams are closed systems with limited opportunities to seek external resources. These studies correspondingly use resource allocation theory to explain *resource losses* associated with engaging in a team activity (i.e., the resources that the team must invest to execute the activity, such as helping behaviors; Barnes et al., 2008). Our study extends this research by highlighting the importance of also considering *resource gains* when using resource allocation theory to examine the consequences of team activities for team resources.

Specifically, we extend the logic of resource allocation theory to include insights from boundary management research. This perspective highlights that teams can realize *resource gains* by engaging in external activities, thus broadening this theory's utility as a conceptual framework for studying the consequences of team activities for team resources and outcomes. Notably, our supplementary results tentatively support this notion, illustrating that boundary management may enhance team performance by increasing the attentional resources available to a team. Given that only a small number of studies have examined the linkage between boundary management and team resources, however, additional research is needed to provide a more robust understanding of the generative mechanisms underlying the team boundary management–performance linkage.

Moreover, we believe that our exploration of methodological moderators offers important insights for boundary management research. For instance, we found higher estimates for effect sizes that used data from the same source to measure the boundary management and outcome variables (which was the case for 41% of the effect sizes in our sample) than for effect sizes calculated with different data sources or objective data. Scholars have noted that same-source designs may induce common-source or method variance and thus produce distorted findings (e.g., Podsakoff et al., 2012). Hence, boundary management researchers would be well advised to carefully consider this issue when designing their studies to avoid undue biases.

In contrast, we did not find different effect sizes between studies applying general boundary management measures (e.g., overall frequency of external communication; Keller, 2001; Scott, 1997) and those capturing more specific boundary management activities (e.g., coordination, representation, or general information search). This finding does not confirm earlier studies advising against the use of general boundary management measures to ensure that the richness of this phenomenon is captured (Ancona & Caldwell, 1992a, 1992b). Furthermore, we found no differences among studies using network or Likert-type measures of boundary management, although we note that network measures may be preferable if researchers desire a more detailed assessment of the configuration of a team's boundary management activities (Carter, Cullen-Lester, Jones, Gerbasi, Chrobot-Mason, & Nae, 2020; Cullen-Lester, Maupin, & Carter, 2017).

Similarly, boundary management measures based on referent-shift or other aggregation models (e.g., direct consensus) yielded comparable effect sizes, indicating that scholars’ adoption of either approach should be driven more by theoretical considerations than empirical considerations (see Chan, 1998; Morgeson & Hofmann, 1999). Moreover, we found equivalent effect sizes for efficiency- and effectiveness-related performance criteria, indicating that boundary management may similarly benefit an array of team outcomes. Finally, our finding of similar effects for team members’ and team leaders’ boundary management suggests that the use of alternative data sources in empirical studies does not lead to different conclusions.

### Practical Implications

Our meta-analysis provides managers with novel insights into how teams’ boundary management can be organized and structured to yield optimal performance advantages. Specifically, our findings illustrate that teams may benefit the most from (a) primarily focusing their boundary management efforts on constituents outside their home organization and (b) emphasizing boundary spanning (e.g., coordination, representation, and information search) rather than boundary strengthening (e.g., buffering, guarding, and sentry activities).

These findings have tangible implications for managerial practice. From a broader organizational perspective, it seems advisable to incorporate boundary management activities into human resource development practices (e.g., team training and development activities; Marrone et al., 2007). Organizations may strive to promote employees’ boundary management behaviors, emphasize the use of boundary-spanning rather than boundary-strengthening approaches, and encourage employees to not limit their boundary management efforts to internal parties but also consider extraorganizational targets. Allowing employees to complete temporary internships in other companies (de Vries et al., 2022) or hiring new employees with broad external networks (Kleinbaum, 2012) may prove useful in this regard. Firms can also reinforce extraorganizational boundary management by engaging in multipartner collaborations and exposing their employees to work within interorganizational alliance teams (Heidl, Steensma, & Phelps, 2014).

Furthermore, prior research has shown that team members often look to their formal leaders as role models and emulate those leaders’ behaviors (e.g., Weaver, Trevino, & Agle, 2005). Hence, formal leaders should emphasize appropriate boundary management activities in their own behavior, encourage their team members to develop and maintain productive relationships with external parties, and venture outside the confines of their organizations when needed. In fact, although somewhat unexpected, our findings illustrate that formal leaders’ boundary management can be particularly effective. Hence, formal leaders should be aware of their crucial role in this regard as direct carriers of their team's boundary management activities and as role models for their team members’ activities.

### Limitations and Directions for Future Research

Our work has several limitations that point to opportunities for future research. Most studies in our sample provided limited information on the target of team boundary management, which restricted our theorizing to the differences between intra- and extraorganizational targets. In reality, these broad categories are likely to encompass variability, which could influence relevant resource losses and gains. For example, with regard to intraorganizational targets, teams on lower hierarchical levels might find it difficult to access upper management, which could increase the costs of communicating with such internal constituents. Conversely, some extraorganizational entities, such as local customers or suppliers, may be relatively easy to access, thereby decreasing the costs of external boundary management. Importantly, however, such instances should work in favor of our hypothesis, moving the trade-off between resource costs and gains in an even more favorable direction for extraorganizational boundary management. Additional research is needed to examine these boundary conditions to our theorizing by using finer-grained categorizations that differentiate between distinct intra- and extraorganizational targets and a more exhaustive measure of organizational size.

Relatedly, it might be particularly interesting to consider how social identity processes affect the benefits of extra- versus intraorganizational boundary management. For extraorganizational boundary management, the parties involved may jointly identify with their overall profession or industry, even though they also identify with their distinct home organizations (Birkinshaw, Ambos, & Bouquet, 2017; Schotter, Mudambi, Doz, & Gaur, 2017). This shared professional or industry identity could provide teams from different organizations with common grounds for effectively exchanging resources, thereby strengthening the benefits of extraorganizational boundary management by reducing the resource investments associated with such activities. With regard to intraorganizational boundary management, the effects of shared identities might be less pronounced, as teams’ compatible goal structures and working methods already enable boundary management processes. As such, shared identities might strengthen the benefits of extra- versus intraorganizational boundary management. We encourage scholars to examine this issue in detail to advance our understanding of the implications of boundary management.

Moreover, we note that the 19 studies on extraorganizational boundary management in our meta-analytic sample exclusively examined teams with project, decision-making, or mixed tasks, whereas none of these studies examined teams with production-planning tasks. Hence, the generalizability of our findings on the target of boundary management should be considered with caution. The benefits of extraorganizational boundary management, as uncovered in our analyses, may primarily apply to teams that critically rely on external ties for effective task completion (e.g., to gather critical information for complex decisions; Keller, 2001). Notably, production-planning teams and teams with similar administrative support functions may require less external boundary management, whereas internal boundary management may be more relevant to them, as these teams are generally focused on routine tasks and internal operations and efficiency (Cagliano, Caniato, & Spina, 2006; De Wit, Greer, & Jehn, 2012). Additional studies are therefore needed to further clarify the roles of intra- versus extraorganizational boundary management for more diverse team types, including those primarily responsible for internal tasks.

Furthermore, our sample included a small number of effect sizes for boundary-strengthening (*n *= 27) relative to boundary-spanning (*n *= 190) activities. This provides rich grounds for future research. For example, we found no study that examined extraorganizational boundary management aimed at strengthening boundaries (e.g., preventing a team from leaking confidential information to external parties). In addition to shedding light on this particular category of boundary management, additional research seems necessary to distinguish the performance consequences associated with specific subtypes of boundary strengthening, such as boundary reinforcement (i.e., keeping team members focused on internal tasks) and boundary buffering (i.e., shielding the team from external constituents; Faraj & Yan, 2009).

Moreover, we cannot draw strong causal inferences from our results because none of the studies in our sample experimentally manipulated team boundary management or applied truly longitudinal designs (i.e., with all focal variables measured at multiple points in time; Shadish, Cook, & Campbell, 2002). While some of the primary studies (*n *= 17) implemented a time lag between their measurements of boundary management and team performance, we did not find significant effect size differences between studies with and without such lags. However, this does not rule out potential reverse or reciprocal causality (see Antonakis, Bendahan, Jacquart, & Lalive, 2010). For example, high-performing teams may have more resources for boundary management. Hence, future research using experimental or longitudinal designs is needed before causality claims can be made.

This lack of longitudinal research also implies that we could not incorporate a temporal perspective into our meta-analysis. Future research could benefit from addressing this issue. Ancona and Caldwell (1988), for instance, offers anecdotal evidence that boundary spanning may be particularly effective in the earlier stages of a project's life cycle when a team is exploring different options and searching for information on how to solve its tasks (see also Hoegl & Weinkauf, 2005). In contrast, boundary strengthening may become more important in later stages because it may help team members focus on task completion and finish a project without external disturbances (Ancona & Caldwell, 1988). Similarly, some boundary activities may be most effective when enacted on a continuous basis (e.g., coordinating with outside parties; Ancona & Caldwell, 1988; Faraj & Yan, 2009), while others may be necessary only in certain situations (e.g., strengthening team boundaries after an intense period of resource acquisition; Yan & Louis, 1999). The incorporation of such temporal considerations may advance a more complete understanding of boundary management's performance implications.

We also note that only one primary study in our meta-analysis examined nonlinear effects (Gibson & Dibble, 2013). Research applying a resource allocation framework to other phenomena has begun to investigate curvilinear relationships (e.g., Mitchell et al., 2016), which could also be interesting for the boundary management literature. Our results illustrate that, in many instances, the benefits of boundary management outweigh its costs. Along these lines, it would be worthwhile to explore whether there is a “too much of a good thing” effect (Pierce & Aguinis, 2011) in which the positive performance consequences of boundary management reverse at some point. Similarly, examining such nonlinear relationships for distinct targets, types, and carriers of boundary management may be worthwhile and could provide additional nuances to the findings presented here.

In addition, whereas our theorizing was based on team-level resource allocation theory, most of the primary studies in our meta-analysis did not examine resource-based mechanisms in relation to the consequences of boundary management, and none of the studies directly tested trade-offs between resources. We therefore encourage further research on the key resource-based mechanisms that allow teams to benefit from their members’ boundary management efforts. Future research may systematically investigate how the trade-off of resources gains or losses by different boundary management carriers, targets, and types may mediate the effect of boundary management on team performance.

More generally, we believe that it would be valuable to extend our team-level resource allocation model with other conceptual perspectives to address the remaining questions associated with team boundary management in an integrative manner. Future research could, for example, combine our conceptual model with insights from resource dependency theory (Pfeffer & Salancik, 1978) to investigate why organizational teams engage in boundary management in the first place. Alternatively, subsequent research could combine our model with insights from resourcing theory (Feldman, 2004) to address the question “What do organizational teams consider to be relevant resources and how do they put such resources to use?”

In sum, we believe that research on boundary management has made important advances in recent decades, as illustrated and summarized in our meta-analysis. At the same time, relevant questions remain, and we hope that this investigation provides a new stimulus for addressing such issues.

## Figures and Tables

**Appendix 2:**
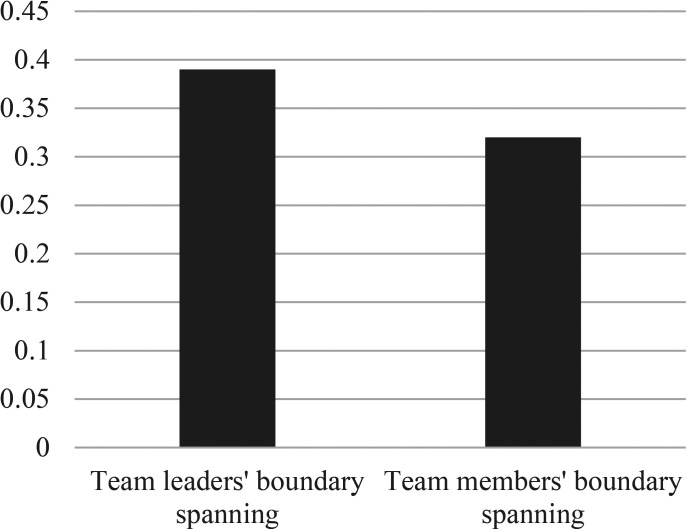
Predicted Effect Sizes (*ŷ*) for Team Leaders’ Versus Team Members’ Boundary Spanning

**Appendix 3:**
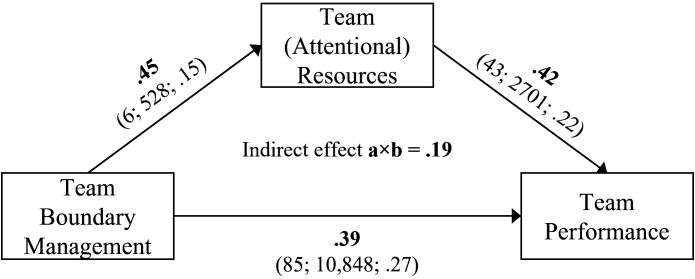
Mediation Analysis of Team Boundary Management, Team (Attentional) Resources, and Team Performance

*Note:* Values in parentheses denote the number of independent studies (*k*), the cumulative sample size (*n*), and standard deviation of corrected correlations (*SD*_ρ_).

## Notes

1. Consistent with prior team research and associated meta-analyses (e.g., Bell, 2007; Greer et al., 2018), we define team performance as the degree to which a team accomplishes its key goals, as reflected by such indicators as output quality, quantity, and efficiency (Devine & Philips, 2001).

2. The studies examining such interactive associations have mainly focused on contextual characteristics. They have, for example, illustrated that boundary management is more effective with nonroutine, complex, or innovative team tasks (Faraj & Yan, 2009; Wu et al., 2020); in volatile, uncertain environments (Gibson & Dibble, 2013); for functionally diverse teams (Cummings, 2004); and for teams that lack cohesion (Carbonell & Rodríguez Escudero, 2019) or enjoy top management support (Sleep et al., 2015). Although interesting, this research does not address the key question underlying the present investigation of how teams can organize their boundary management activities to be most effective.

3. Resource allocation theory provides a relatively narrow understanding of resources. It focuses more on attentional or cognitive resources than on material or financial resources (Kanfer & Ackerman, 1989). The information-processing perspective suggests that these attentional resources are limited (Kanfer & Ackerman, 1989) and that individuals’ performance depends on the cognitive effort they devote to a task.

4. We considered other operationalizations of the performance criterion (e.g., by differentiating between efficiency- and innovation/creativity-related measures), but these analyses did not change the conclusions of our results.

5. Additionally, we performed bivariate analyses of the mean effect sizes for primary studies using Likert-type measures (*k *= 70; ρ* *= 0.43, 95% CI [0.36, 0.49]) versus network measures of boundary management (*k *= 8; ρ* *= 0.22, 95% CI [0.04, 0.40]). Although the confidence intervals for these effect size estimates overlap (suggesting that they are not significantly different), we note that the absolute effect size difference is rather substantial. Hence, some caution is warranted regarding whether Likert-type versus network measures of boundary management indeed capture identical constructs. Future research should examine this issue and focus on the relatively underexplored linkage between network measures of boundary management and team performance.

6. Although the measurement error of team-level means contains item- and rater-specific error variance, the latter is often the main source of measurement error. As such, ICC_2_ reliabilities are the more appropriate reliability statistic for correcting study correlations (De Jong et al., 2016; Greer et al., 2018). However, as we do not correct for item-specific measurement error, our artifact distribution corrections are incomplete. Our results should therefore be understood as conservative estimates of the true population correlations (De Jong et al., 2016; Greer et al., 2018).

7. The formula for this large-sample approximation is based on the following equation (see Borenstein, 2009: 321, equation 12.27), with *r* indicating a raw correlation coefficient and *n* representing the sample size:

$v_{r}=\;\frac{{\left(1-r^{2}\right)}^{2}}{n-1}.$

8. The sample size for each moderator is noted in Table 2. The fourth column (i.e., “Effect sizes, *k*”) shows the number of effect sizes coded for each category. The overall sample size for the dichotomous moderators is the sum of both categories.

9. One could argue that sales teams have stronger extraorganizational ties than other teams. As such, sales teams may drive the greater effect size of extraorganizational boundary management relative to intraorganizational boundary management. In our meta-analytic data set, only one study examined a primary sample of 167 sales teams (Sleep et al., 2015). After controlling for those sales teams (i.e., by coding the effect sizes of all other studies [*k *= 289] vs. the effect sizes of this study of sales teams [*k *= 6]), the conclusions of our results did not change for extra-/intraorganizational boundary management or any other study variables.

10. To offer an illustration of the practical relevance of our findings, we calculated the predicted effect sizes for the conditions of our theoretical moderators. To do so, we inserted the values of the conditions of the moderators (i.e., 0 and 1) into our regression equation and imputed the intercept with the sample average of ρ* *= 0.39, as our multilevel meta-regression algorithm constraints the intercept by default at 0 (Van den Noortgate et al., 2014). By taking the other theoretical moderators and the control variables into account, the predicted boundary management–team performance effect sizes were *ŷ *= 0.39 for team leaders’ and *ŷ *= 0.13 for team members, *ŷ *= 0.39 for intraorganizational boundary management and *ŷ *= 0.48 for extraorganizational boundary management, and *ŷ *= 0.39 for boundary-strengthening activities and *ŷ *= 0.57 for boundary-spanning activities. Although these predicted effect sizes can offer a coarse-grained illustration of the relative sizes of the effects, they cannot be regarded as an exact representation of the effects, as our analysis cannot provide precision to such an extent.

11. A direct examination of this conclusion in the present data is aggravated by the fact that our sample studies included very few effect sizes for boundary-strengthening activities among leaders (*k *= 1) and team members (*k *= 4), because studies on boundary strengthening did not often specify the carrier. Nevertheless, we conducted an exploratory analysis of the full interactive relationship of boundary-spanning type (i.e., spanning vs. strengthening) and carrier (i.e., leaders vs. members) with team performance. We again found a significant Carrier × Type interaction (γ* *= 0.20, *SE *= .09, *p *= .002). Moreover, the predicted effect sizes for boundary-spanning activities were *ŷ *= 0.43 among leaders and *ŷ *= 0.30 among members, whereas the predicted effect sizes for boundary-strengthening activities were *ŷ *= 0.39 among leaders and *ŷ *= 0.06 among members. Although these results should be interpreted with caution, they are generally consistent with our previous conclusion.

## Appendix

**Appendix 1:** Overview of Coding Criteria for the Contingency Factors

Contingency Factor: Moderator Values	Coding Criteria	Exemplary Studies
**Carrier of boundary management**		
Formal leader (0)	Boundary management items solely refer to the leader, administrator, manager, or supervisor of the team	Davison et al. (2012), Marks et al. (2005), Richter et al. (2006)
Team members (1)	Boundary management items refer to the members of a team	Gibson and Dibble (2013), Faraj and Yan (2009), De Vries et al. (2016), Marrone et al. (2007)
**Target of boundary management**		
Intraorganizational (0)	When the focal team engages in boundary management with entities from inside its home organization	De Vries et al. (2014), Oh et al. (2004), Richter et al. (2006)
Extraorganizational (1)	When the focal team engages in boundary management with entities from outside its home organization	Gopal and Gosain (2010), Katz (1982), Mishra and Shah (2009)
**Type of boundary management**		
Boundary strengthening (0)	When the majority of items resemble one or more of the following items:	
Sentry activities	• Absorb outside pressures for the team so it can work without interference • Protect the team from team-external interference • Prevent other actors from “overloading” the team with too much information or too many requests	Faraj and Yan (2009), Sawyer et al. (2010), Somech and Khalaili (2014)
Guard activities	Avoid the release of relevant team-internal information	Guinan et al. (1998), Lee et al. (2017), Sawyer et al. (2010)
Boundary spanning (1)	When the majority of items resemble one or more of the following items:	
Coordination	• Resolve problems with team-external groups • Coordinate activities with team-external groups • Negotiate with others about work-related issues • Review products/services/work with outsiders	Ancona and Caldwell (1992a, 1992b), DeChurch and Marks (2006), de Vries et al. (2016)
Representation	• Persuade others to support the team's decisions • Acquire resources (money, new members, equipment) for the team • Report the team's progress to a higher organizational level • Find out whether influential team-external actors support or oppose the team's activities • Obtain information on the company's strategy or political situation that may affect the project • Keep other groups in the company informed of the team's activities	Ancona and Caldwell (1992a, 1992b), Edmondson (1999), Marrone et al. (2007)
General information search	• Find out what competing firms or groups are doing • Scan the intra- or extraorganizational environment for ideas/expertise • Collect information/ideas from individuals outside the team • Scan the intra- or extraorganizational environment for ideas/expertise	Ancona and Caldwell (1992a, 1992b), Haas (2010), Keller (2001)
Knowledge/resource exchange	• Exchange important information (e.g., market trends, sources of supplies, or ideas for product development) • Offer products or services to other entities • Send team members to other entities to work with them on a joint project	Cummings (2004), Edmondson (2003), Morgan and Piercy (1998)
General boundary management	When the majority of items ask respondents to report on positive work-related interactions with team-external actors without specifying the function of those interactions (e.g., coordination, representation, or information search) or including items that correspond to multiple purposes (e.g., coordination, representation, or information search)	Denison, Hart, and Kahn (1996), Gibson and Dibble (2013), Gladstein (1984)

**Appendix 4:** Meta-Analytic Regression Results (Tests of Hypotheses 2-4) Based on Uncorrected Observed Effect Sizes

Variable^b^	Boundary Management and Team Performance^a^					
	Model 1: Controls Only		Model 2: Research Model		Model 3: Research Model Including Carrier's Spanning	
	γ	*SE*, *p*	γ	*SE*, *p*	γ	*SE*, *p*
**Level 3: Between studies**						
Controls						
Industry	.00	.06, .984	.01	.07, .929	.00	.07, .955
MNC	.00	.06, .998	−.02	.05, .757	−.01	.05, .810
R&D team	−.04	.05, .508	−.06	.05, .273	−.06	.05, .266
Project task	.05	.15, .735	.07	.15, .654	.06	.14, .671
Decision-making task	−.09	.07, .215	−.13	.08, .099	−.13	.08, .095
Production task	−.03	.06, .626	−.02	.06, .721	−.03	.07, .643
Study setting	.03	.09, .774	.05	.10, .639	.05	.10, .638
Publication status	.01	.07, .914	.00	.07, .984	.01	.07, .895
Year of publication	.00	.00, .248	.00	.00, .246	.00	.00, .255
**Level 2: Between effect sizes**						
Methodological moderators						
Boundary management specificity	.03	.03, .336	.03	.03, .351	.04	.03, .223
Team performance criterion	−.02	.03, .445	−.02	.03, .410	−.02	.03, .507
Same measurement source	.08	.03, .018	.07	.03, .013	.07	.03, .017
Network measure	−.17	.12, .152	−.16	.11, .142	−.16	.11, .148
Aggregation model	.05	.08, .510	.07	.08, .391	.06	.07, .388
Respondents	.04	.03, .195	.07	.03, .010	.05	.03, .045
Theoretical moderators						
Carrier: H2			−.13	.06, .034	−.17	.06, .007
Target: H3			.06	.03, .043	.06	.02, .017
Type: H4			.14	.04, .000	.13	.04, .000
Carrier's spanning					.14	.05, .006
**Level 1: Variance within effect sizes**	.02	.01, .007	.01	.01, .034	.01	.01, .152
Overall observations, *n*						
Independent studies	85		85		85	
Effect sizes	295		295		295	
Intraclass coefficient	.36		.36		.36	
Model deviance	4,832.76		5,296.85		5,350.24	

*Note:* Maximum likelihood estimation with robust *SE* and exact *p* value. Entries corresponding to the moderating variables are estimations of the random effects (γ). MNC = multinational company; R&D = research and development.

a Effect sizes are based on uncorrected observed correlations (*r*).

b Coding: industry (0 = manufacturing based, 1 = service based); MNC (0 = no MNC, 1 = MNC); R&D team (0 = no R&D team, 1 = R&D team); project team tasks (0 = no project team tasks, 1 = project team tasks); decision-making team tasks (0 = no decision-making team tasks, 1 = decision-making team tasks); production-planning team tasks (0 = no production-planning team tasks, 1 = production-planning team tasks); study setting (0 = field study, 1 = laboratory study); publication status (0 = unpublished, 1 = published); boundary management specificity (0 = specific boundary management, 1 = general boundary management); team performance criterion (0 = efficiency-related performance criterion, 1 = effectiveness-related performance criterion); same measurement source (0 = independent data sources, 1 = same subjective data source for independent and dependent variables); network measure (0 = Likert-scale measure, 1 = network measure); aggregation model (0 = referent-shift consensus model, 1 = direct consensus model); carrier (0 = formal leader, 1 = team members); target (0 = intraorganizational, 1 = extraorganizational); type (0 = boundary strengthening, 1 = boundary spanning).

**Appendix 5:** Meta-Analytic Regression Results for Different Sets of Control Variables

Variable^b^	Model 1a: Controls Related to Carrier^a^		Model 1b: Controls Related to Target^a^		Model 1c: Controls Related to Type^a^		Model 2: Controls Related to Team Performance^a^		Model 3: With Method Controls^a^		Model 4: No Controls^a^	
	*B*	*SE*, *p*	*B*	*SE*, *p*	*B*	*SE*, *p*	*B*	*SE*, *p*	*B*	*SE*, *p*	*B*	*SE*, *p*
**Level 3: Between studies**												
Controls												
Industry	.08	.10, .426	.05	.10, .569	.07	.10, .467						
MNC	−.03	.08, .740	−.03	.08 .717	−.02	.08, .797						
R&D team	−.07	.08, .392	−.08	.08, .311								
Project task	.23	.20, .247	.17	.21, .407	.27	.22, .219	.25	.22, .266				
Decision-making task	−.15	.08, .050	−.16	.12, .184	−.12	.10, .226						
Production task												
Mixed task			.07	.10, 493	.06	.10, 547						
Methodological moderators												
Study setting			−.01	.14, .938								
Publication status	.03	.11, .823	.00	.11, .975								
Year of publication	.00	.00, 340	.00	.00, .243			.00	.00, .598				
**Level 2: Between effect sizes**												
Methodological moderators												
Boundary management specificity			.06	.04, .164					.08	.04, .057		
Team performance criterion	−.04	.05, .404					−.03	.05, .557	−.03	.04, .459		
Same measurement source			.11	.04, .015	.10	.04, .022	.10	.04, .029	.10	.05, .033		
Network measure			−.26	.15, .090			−.27	.18, .143	−.26	.16, .102		
Aggregation model			.11	.12, .375			.04	.12, .720	.05	.11, .641		
Respondents	.12	.06, .036	.12	.05, .007					.12	.05, .012		
Theoretical moderators												
Carrier: H2	−.31	.10, .001	−.34	.10, .000	−.32	.10, .001	−.29	.10, .004	−.29	.10, −.002	−.28	.10, .005
Target: H3	.08	.03, .015	.09	.04, .013	.08	.04, .017	.06	.04, .079	.08	.04, .026	.07	.03, .038
Type: H4	.18	.06, .004	.16	.06, .007	.16	.06, .011	.16	.06, .009	.15	.06, .010	.17	.06, .009
Carrier's spanning	.28	.08, .000	.26	.08, .001	.30	.08, .000	.29	.09, .001	.25	.09, .003	.32	.08, .000
**Level 1: Variance within effect sizes**	.03	.01, .008	.03	.01, .016	.03	.01, .014	.03	.01, .017	.03	.01, .008	.04	.01, .010
Overall observations, *n*												
Independent studies	85		85		85		85		85		85	
Effect sizes	295		295		295		295		295		295	
Intraclass coefficient	.34		.34		.34		.34		.34		.34	
Model deviance	5,477.14		5,477.14		2,580.00		5,061.98		3,571.69		2,122.10	

*Note:* Maximum likelihood estimation with robust *SE* and exact *p* value. Entries corresponding to the moderating variables are estimations of the random effects (γ). MNC = multinational company; R&D = research and development.

a Effect sizes are based on corrected correlations (ρ).

b Coding: industry (0 = manufacturing based, 1 = service based); MNC (0 = no MNC, 1 = MNC); R&D team (0 = no R&D team, 1 = R&D team); project team tasks (0 = no project team tasks, 1 = project team tasks); decision-making team tasks (0 = no decision-making team tasks, 1 = decision-making team tasks); production-planning team tasks (0 = no production-planning team tasks, 1 = production-planning team tasks); study setting (0 = field study, 1 = laboratory study); publication status (0 = unpublished, 1 = published); boundary management specificity (0 = specific boundary management, 1 = general boundary management); team performance criterion (0 = efficiency-related performance criterion, 1 = effectiveness-related performance criterion); same measurement source (0 = independent data sources, 1 = same subjective data source for independent and dependent variables); network measure (0 = Likert scale measure, 1 = network measure); aggregation model (0 = referent-shift consensus model, 1 = direct consensus model); carrier (0 = formal leader, 1 = team members); target (0 = intraorganizational, 1 = extraorganizational); type (0 = boundary strengthening, 1 = boundary spanning).

**Appendix 6:** Meta-Analytic Regression Results Without Social Network Studies

Variable^b^	Boundary Management and Team Performance^a^					
	Model 1: Controls Only		Model 2: Research Model		Model 3: Research Model Including Carrier's Spanning	
	γ	*SE*, *p*	γ	*SE*, *p*	γ	*SE*, *p*
**Level 3: Between studies**						
Controls						
Industry	.03	.10, .763	.06	.10, .574	.05	.10, .645
Multinational company	.00	.09, .990	−.04	.08, .658	−.03	.08, .758
R&D team	−.01	.08, .909	−.06	.09, .481	−.06	.08., .504
Project task	.05	.22, .816	.08	.22, .717	.07	.20, .737
Decision-making task	−.18	.11, .119	.29	.13, .028	−.28	.13, .032
Production task	−.10	.09, .283	−.09	.09, .322	−.10	.10, .287
Study setting	.19	.10, .059	.25	.13, .053	.24	.12, .051
Publication status	.00	.10, .966	−.03	.10, .804	.00	.10, .978
Year of publication	.01	.00, .140	.01	.00, .109	.00	.00, .109
**Level 2: Between effect sizes**						
Methodological moderators						
Boundary management specificity	.01	.04, .821	.02	.04, .709	.03	.04, .460
Team performance criterion	−.04	.05, .436	−.03	.05, .442	−.03	.05, .570
Same measurement source	.11	.05, .025	.11	.05, .012	.10	.04, .019
Aggregation model	.07	.14, .608	.11	.13, .408	.10	.12, .379
Respondents	.09	.06, .099	.16	.05, .003	.13	.05, .009
Theoretical moderators						
Carrier: H2			−.27	.12, .019	−.33	.11, .002
Target: H3			.10	.05, .038	.10	.04, .013
Type: H4			.17	.07, .009	.16	.06, .010
Carrier's spanning					.26	.10, .006
**Level 1: Variance within effect sizes**	.06	.02, .001	.05	.01, .001	.03	.01, .015
Overall observations, *n*						
Independent studies	72		72		72	
Effect sizes	269		269		269	
Intraclass coefficient	.34		.34		.34	
Model deviance	4,640.49		5,077.01		5,122.56	

*Note:* Maximum likelihood estimation with robust *SE* and exact *p* value. Entries corresponding to the moderating variables are estimations of the random effects (γ). MNC = multinational company; R&D = research and development.

a Effect sizes are based on corrected correlations (ρ).

b Coding: industry (0 = manufacturing based, 1 = service based); MNC (0 = no MNC, 1 = MNC); R&D team (0 = no R&D team, 1 = R&D team); project team tasks (0 = no project team tasks, 1 = project team tasks); decision-making team tasks (0 = no decision-making team tasks, 1 = decision-making team tasks); production-planning team tasks (0 = no production-planning team tasks, 1 = production-planning team tasks); study setting (0 = field study, 1 = laboratory study); publication status (0 = unpublished, 1 = published); boundary management specificity (0 = specific boundary management, 1 = general boundary management); team performance criterion (0 = efficiency-related performance criterion, 1 = effectiveness-related performance criterion); same measurement source (0 = independent data sources, 1 = same subjective data source for independent and dependent variables); network measure (0 = Likert scale measure, 1 = network measure); aggregation model (0 = referent-shift consensus model, 1 = direct consensus model); carrier (0 = formal leader, 1 = team members); target (0 = intraorganizational, 1 = extraorganizational); type (0 = boundary strengthening, 1 = boundary spanning).
